# Ammonia Oxidation Property of Soils on the Glacier Foreland of Austre Brøggerbreen, Svalbard: Response to Open-top Chamber Experiments

**DOI:** 10.1264/jsme2.ME25058

**Published:** 2025-11-20

**Authors:** Kentaro Hayashi, Keisuke Ono, Yukiko Tanabe, Masahito Hayatsu, Kanako Tago, Tsubasa Ohbayashi, Yong Wang, Luciano Nobuhiro Aoyagi, Masaki Uchida

**Affiliations:** 1 Research Institute for Humanity and Nature, National Institutes for the Humanities, Kyoto 603–8047, Japan; 2 Institute for Agro-Environmental Sciences, National Agriculture and Food Research Organization, Tsukuba 305–8604, Japan; 3 National Institute of Polar Research, Tachikawa 190–8518, Japan; 4 School of Veterinary Medicine, Kitasato University, Sagamihara 252–0373, Japan; 5 TanBIO Inc., Tsukuba 305–0035, Japan; 6 SOKENDAI (The Graduate University for Advanced Studies), Tachikawa 190–8518, Japan

**Keywords:** ammonia oxidation potential, ammonia-oxidizing bacteria, high arctic, nitrification, passive heating

## Abstract

Recent warming and glacier retreat in the Svalbard Archipelago, part of the Arctic cryosphere, have become increasingly evident. The present study investigated the foreland of Austre Brøggerbreen near Ny-Ålesund to clarify how soil nitrification responds to changing conditions. Two sites, exposed for different periods following glacier retreat, were compared. A manipulation experiment using open-top chambers (OTCs) and homogenized initial soil conditions was conducted to assess the effects of site differences, the OTC treatment, soil depth, and interannual variations on soil and nitrification properties. Although the OTC treatment slightly increased soil temperature and moisture, its overall effect on soil properties, ammonia oxidation potential (AOP), and microbial properties was negligible. In contrast, homogenization markedly increased total nitrogen at both sites and temporarily boosted AOPs for two years before levels returned to baseline. Site 2, exposed for longer, contained more soil nitrogen and showed higher AOP than Site 1. For example, in 2015, AOPs at 10°C were 3.5 and 2.4‍ ‍ng N g^–1^ dry soil h^–1^ at Sites 2 and 1, respectively. Ammonia-oxidizing bacteria (AOB) and archaea (AOA) were both more abundant at Site 2, although AOB clearly dominated at both sites. While AOB-*amo*A operational taxonomic units (OTUs) were mostly shared between sites, community compositions differed: OTU2 was prevalent at Site 1, but minor at Site 2. OTU2 may act as a pioneer taxon that declines in later stages, or its pattern may reflect site-specific soil conditions. A phylogenetic anal­ysis showed that OTU2 and OTU3 belonged to Cluster ME found near Mount Everest.

Arctic islands, such as the Svalbard Archipelago, are characterized by harsh cryospheric conditions with glaciers, permafrost, and tundra ecosystems that have low biodiversity and simple structures. Despite this, they form heterogeneous environments where terrestrial, marine, freshwater, and glacial systems are closely linked (Pedersen *et al.*, 2022). Svalbard is one of the fastest warming regions worldwide: from 1971 to 2017, mean air temperatures rose by 0.71°C per decade, with strong winter warming by 1.35°C in some cases (Pedersen *et al.*, 2022). While most warming occurred in winter, summer temperatures have also increased steadily since 1980, with recent years setting new records, and 2024 marking the highest mean summer and August temperatures on record (van den Broek *et al.*, 2025). This rapid warming accelerates snow and ice melt. Although glacier retreat has long been observed in Svalbard, its recent acceleration is notable. Li *et al.* (2025) showed that the retreat of 149 marine-terminating glaciers from 1985 to 2023 was highly sensitive to atmospheric and oceanic warming, with continued warming likely to intensify future glacier loss.

The terminus of a glacier on land is called the glacier front, and the newly exposed area is the glacier foreland, where soil formation resumes. The warming-driven retreat and thinning of glaciers expose new forelands where soils, vegetation, and microbial communities undergo primary succession, reshaping hydrology and ecosystems (Pedersen *et al.*, 2022). In Arctic regions, biological production is slow, and pioneer plant establishment takes decades; decomposition is even slower, allowing the gradual accumulation of organic matter and nitrogen (N) (Callaghan *et al.*, 2005; Bardgett *et al.*, 2007). Austre Brøggerbreen (East Brøgger Glacier) near Ny-Ålesund has significantly retreated, with studies investigating carbon (C) and N cycling on its foreland. Early-stage soil crusts stabilize surfaces and enrich soil‍ ‍nutrients (Nakatsubo *et al.*, 2005). Near Ny-Ålesund, cyanobacteria in soil crusts play a key role in biological N‍ ‍fixation, driving N cycles (Hodkinson *et al.*, 2003; Yoshitake *et al.*, 2010; Wietrzyk-Pełka *et al.*, 2018). Older forelands generally hold more microbial biomass C and N, and later successional stages support more diverse communities with coexisting bacteria and fungi (Yoshitake *et al.*, 2018).

Nitrification, the only soil process producing nitrate via nitrite, occurs even in soils where mean temperatures remain below 0°C (Banerjee and Siciliano, 2012; Alves *et al.*, 2013; Hayashi *et al.*, 2016, 2020). For example, climax-stage *Salix polaris*–moss soils in Ny-Ålesund were found to have high ammonia oxidation potentials (AOPs) (Hayashi *et al.*, 2016). However, nitrification in newly exposed forelands has not been exami­ned in detail. It currently remains unclear how nitrifiers develop in these soils and also how they respond to warming.

To address this, three approaches are commonly used: incubation experiments with collected samples (Yoshitake *et al.*, 2007; Hayashi *et al.*, 2014); chronosequence studies comparing sites with different exposure ages (Hodkinson *et al.*, 2003; Yoshitake *et al.*, 2018; Kim *et al.*, 2022); and *in situ* manipulations using open-top chambers (OTCs) or snow fences (Wahren *et al.*, 2005; Lupascu *et al.*, 2018; Hollister *et al.*, 2022). Laboratory incubation experiments do not accurately represent *in situ* conditions, while the chronosequence approach assumes that differences among sites reflect only temporal variations, an assumption that may not hold due to site-specific environmental factors. Therefore, in the present study, we selected the OTC method to directly assess warming responses under field conditions. We herein investigated the nitrification properties of soils in the foreland of Austre Brøggerbreen using a passive warming experiment with OTCs at two sites that differed in years since deglaciation.

## Materials and Methods

### Research sites

**Fig. 1.**
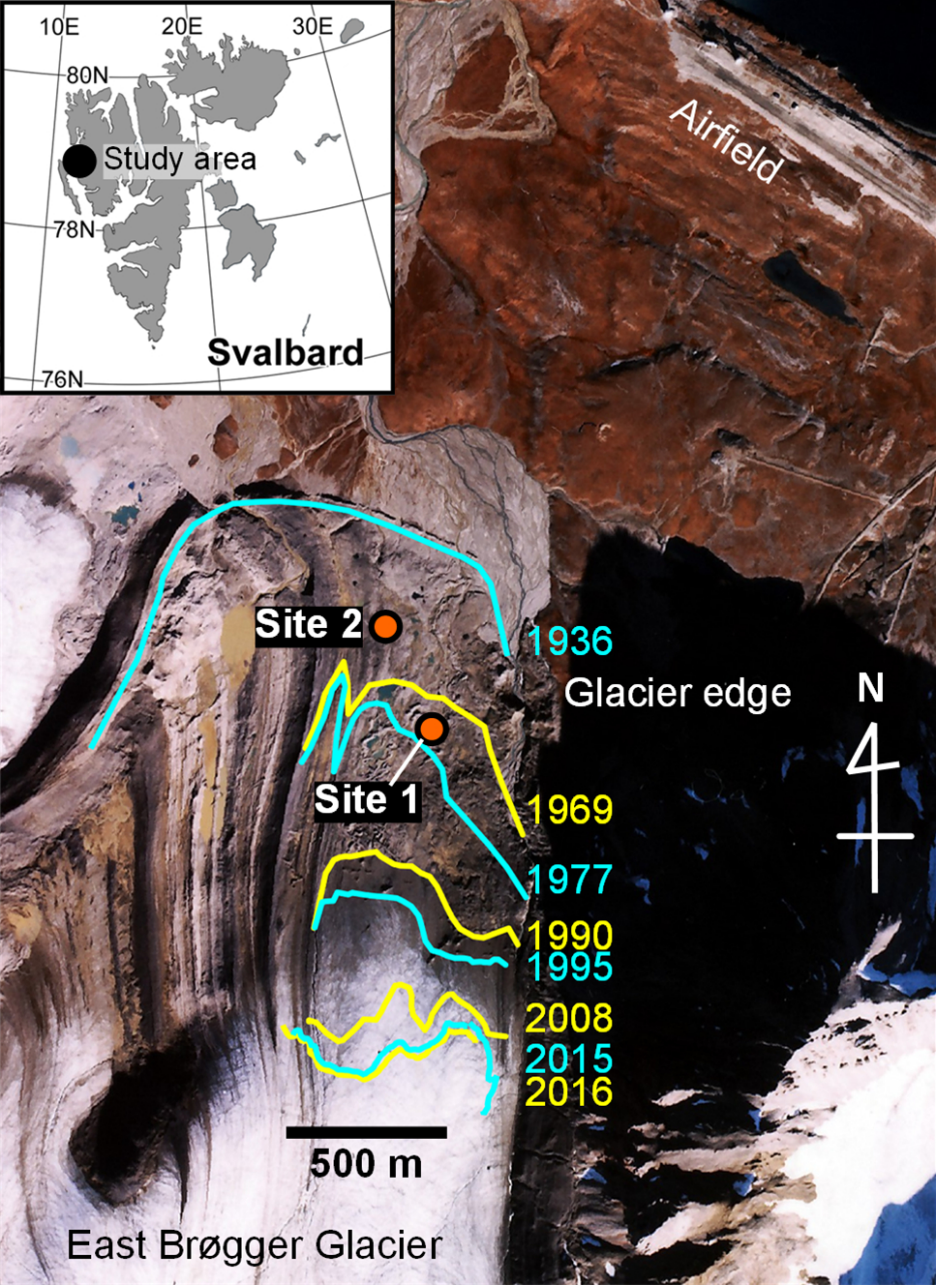
Research sites located in the glacier foreland of Austre Brøggerbreen (East Brøgger Glacier), Svalbard. The aerial image was provided by the Norwegian Polar Institute.

The study area was the foreland of Austre Brøggerbreen, Svalbard (Fig. 1), where glacier retreat has continued since the 20th century. Research was conducted at two of the four permanent exclosures established by the National Institute of Polar Research, Japan in 1994: Site 1 (78°54.76′N, 11°50.03′E) and Site 2 (78°54.91′N, 11°49.63′E) (Fig. 1). These sites represent earlier stages of vegetative succession after glacier retreat (Yoshitake *et al.*, 2018) and are approximately 350‍ ‍m apart. Aerial photographs indicate that ice coverage disappeared between 1969 and 1977 at Site 1 and between 1936 and 1969 at Site 2. Additional site information is shown in the Supplementary Materials (Fig. S1).

### Field experiments

**Fig. 2.**
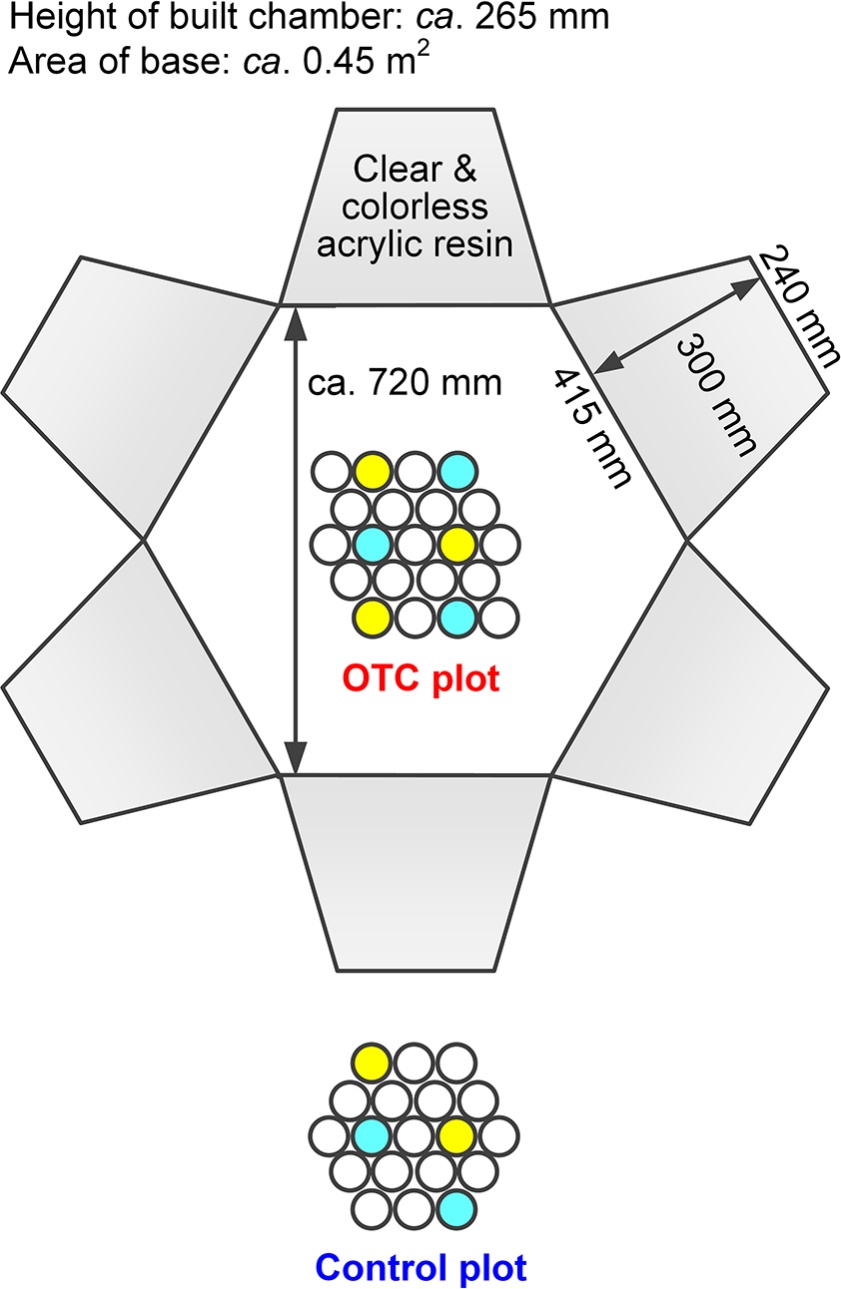
Composition of the open-top chamber. Yellow and blue circles denote soil cores for monitoring systems 1 and 2, respectively. Each monitoring system consisted of one datalogger and five sensors (temperature and volumetric water content), three of which were installed at the open-top chamber plot and the other two sensors at the control plot, to minimize the risk of missing data due to sensor or data logger failure.

A passive soil warming experiment using OTCs was conducted at both sites from July 24, 2015 to July 16, 2019 (Site 1) and from July 27, 2015 to July 16, 2019 (Site 2). Each site had an OTC plot and uncovered control (CTL) plot. Soil collected nearby was sieved, mixed to homogenize conditions, and filled into polyvinylchloride (PVC) cores. Some cores were used for continuous soil temperature and moisture monitoring; others were collected every summer in triplicate for anal­yses. Each of the collected cores was divided into topsoil (0–2‍ ‍cm) and subsoil (>2‍ ‍cm). Each OTC consisted of six colorless trapezoidal acrylic panels (top 240‍ ‍mm, bottom 415‍ ‍mm, height 300‍ ‍mm, and thickness 3‍ ‍mm) forming a hexagonal enclosure (Fig. 2). Assembly details, PVC core specifications, and the soil collection method are provided in the Supplementary Materials (Fig. S2 and S3). Samples were stored at approximately 4°C until transported to the laboratory in Japan. Subsamples for genetic anal­yses were stored at –80°C until analyzed.

At each site, soil temperature and moisture sensors (5TM; Decagon) were installed in six OTC and four CTL cores. The ten sensors were connected to two dataloggers (Em50; Decagon). To reduce the risk of data loss, sensors were split between loggers (Fig. 2). Additional set-up and maintenance details are included in the Supplementary Materials (Fig. S2).

### Soil anal­ysis

Measurements included pH (H_2_O), total nitrogen (TN), total carbon (TC), the CN ratio (w/w), and inorganic N (ammonium, nitrate, and nitrite) (*n*=3) for initial homogenized soils (2015, two sites) and annual soil samples (2016–2019, two sites, OTC/CTL, topsoil/subsoil). Fresh soil pH (H_2_O) was measured with a pH meter (B-712; Horiba). Other properties were assessed using air-dried soil. Moisture content was evaluated using a drying oven. TN and TC were analyzed with an NC analyzer (Sumigraph NC-22F; Sumika Chemical Analysis Service). Inorganic N was measured with a flow injection analyzer (AQLA-1000; Aqualab) after extraction from 4‍ ‍g of air-dried soil with 10‍ ‍mL of 10% potassium chloride.

### Microbial anal­ysis

AOPs, *amo*A gene copy numbers of ammonia-oxidizing bacteria (AOB) and archaea (AOA), and AOB-*amo*A operational taxonomic units (OTUs) were analyzed. A phylogenetic tree including major AOB-*amo*A OTUs was also constructed. AOPs were analyzed for annual samples, whereas other microbial parameters were analyzed for samples from 2015, 2017, and 2019. Further details are provided in the Supplementary Materials.

AOPs were assessed by measuring nitrite production under excess substrate (*n*=3). Fresh soil (2.5 g, 2-mm sieve) was incubated with 1‍ ‍mM ammonium sulfate, 10‍ ‍mM sodium chlorate to inhibit nitrite oxidation (Belser and Mays, 1980), and a phosphate buffer (pH 7) at 10°C and 20°C for 24‍ ‍h with shaking. Nitrite was measured colorimetrically.

Soil DNA (*n*=3) was extracted with the FastDNA Spin Kit for Soil (Qbiogene/MP Biomedicals) and purified. AOB- and AOA-*amo*A gene copy numbers were quantified by SYBR Green I-based real-time PCR with established primers and conditions (Rotthauwe *et al.*, 1997; Avrahami *et al.*, 2003; Tourna *et al.*, 2008; Morimoto *et al.*, 2011; Levičnik-Höfferle *et al.*, 2012; Shimomura *et al.*, 2012).

To assess the diversity of AOB-*amo*A, high-throughput sequencing targeting the *amo*A gene was conducted. PCR amplification for AOB-*amo*A was performed using the primer set *amo*A-1F/*amo*A-2R-GG (Nicolaisen and Ramsing, 2002). Amplicons were indexed with unique 8-bp barcodes, pooled in equimolar concentrations, and sequenced on an Illumina MiSeq platform (2×300 bp; Illumina). Sequencing was outsourced to Bioengineering Lab.

Raw sequencing reads for AOB-*amo*A were processed using DADA2 (v1.10.1; Callahan *et al.*, 2016) for quality filtering, denoising, dereplication, and chimera removal. Reads were trimmed to 290 bp (forward) and 215 bp (reverse) to exclude low-quality regions. The resulting sequences were clustered into OTUs at 95% similarity using Mothur (v1.42.2; Schloss *et al.*, 2009), following the approach described by Tago *et al.* (2015). Alpha diversity metrics were calculated based on OTU tables using Mothur.

Taxonomic assignments of representative sequences were made via local BLASTN searches against *amo*A reference sequences from the National Center for Biotechnology Information database using BLAST+. Multiple sequence alignments of the AOB-*amo*A gene were conducted using MAFFT (Katoh *et al.*, 2002), and a maximum likelihood phylogenetic tree was constructed in MEGA (v10.1.8; Kumar *et al.*, 2018; Stecher *et al.*, 2020) with 1,000 bootstrap replicates. The Tamura-Nei substitution model with a gamma distribution and invariant sites (G+I) was applied (Tamura and Nei, 1993).

### Statistical anal­ysis

Descriptive statistics and initial anal­yses of chemical and microbial data were performed using the SAS Add-In for Microsoft Office (SAS Institute). Differences in AOB α-diversity indices among samples were tested using an anal­ysis of variance (ANOVA) and paired *t*-tests in R. To assess the effects of environmental factors on the AOB community structure, a PERmutational Multivariate ANalysis Of Variance, two-way ANOVA (PERMANOVA) was conducted using the adonis package in R (R Foundation for Statistical Computing). Variation partitioning anal­yses were performed using the vegan package in R.

## Results

### Soil temperature and moisture

Soil temperature and moisture were recorded continuously throughout the 4-year study. Measurements were taken every 4‍ ‍h and averaged daily. The overall means for the study period are shown in Table 1. In the CTL plots, mean soil temperatures were –0.69°C at Site 1 and –1.58°C at Site 2, with volumetric water contents of 0.099 and 0.094 (equivalent to 9.9% and 9.4% by volume), respectively. Daily values are provided in the Supplementary Materials (Fig. S4).

At each site, paired daily mean values for the OTC and CTL plots were compared using a *t*-test. OTC plots showed significantly higher soil temperatures, with warming effects of 0.39°C at Site 1 and 0.56°C at Site 2 (*P*<0.05, Table 1). Although volumetric water content was also significantly higher in the OTC plots, the difference from the CTL plots was small at 0.002–0.003 (0.2%–0.3%).

### Soil properties

Averaged across all plots, soil depths, and years, soils were alkaline, with pH values of 9.3 at Site 1 and 8.8 at Site 2. TC, TN, and the CN ratio were 0.11%, 0.0054%, and 21.0, respectively, at Site 1 and 0.35%, 0.026%, and 13.6, respectively, at Site 2. Ammonium and nitrate contents were 0.66 and 0.63‍ ‍μg N g^–1^ dry soil, respectively, at Site 1 and 2.4 and 0.70‍ ‍μg N g^–1^ dry soil, respectively, at Site 2. Nitrite levels were negligible. Detailed soil data are shown in the Supplementary Materials (Table S1).

**Fig. 3.**
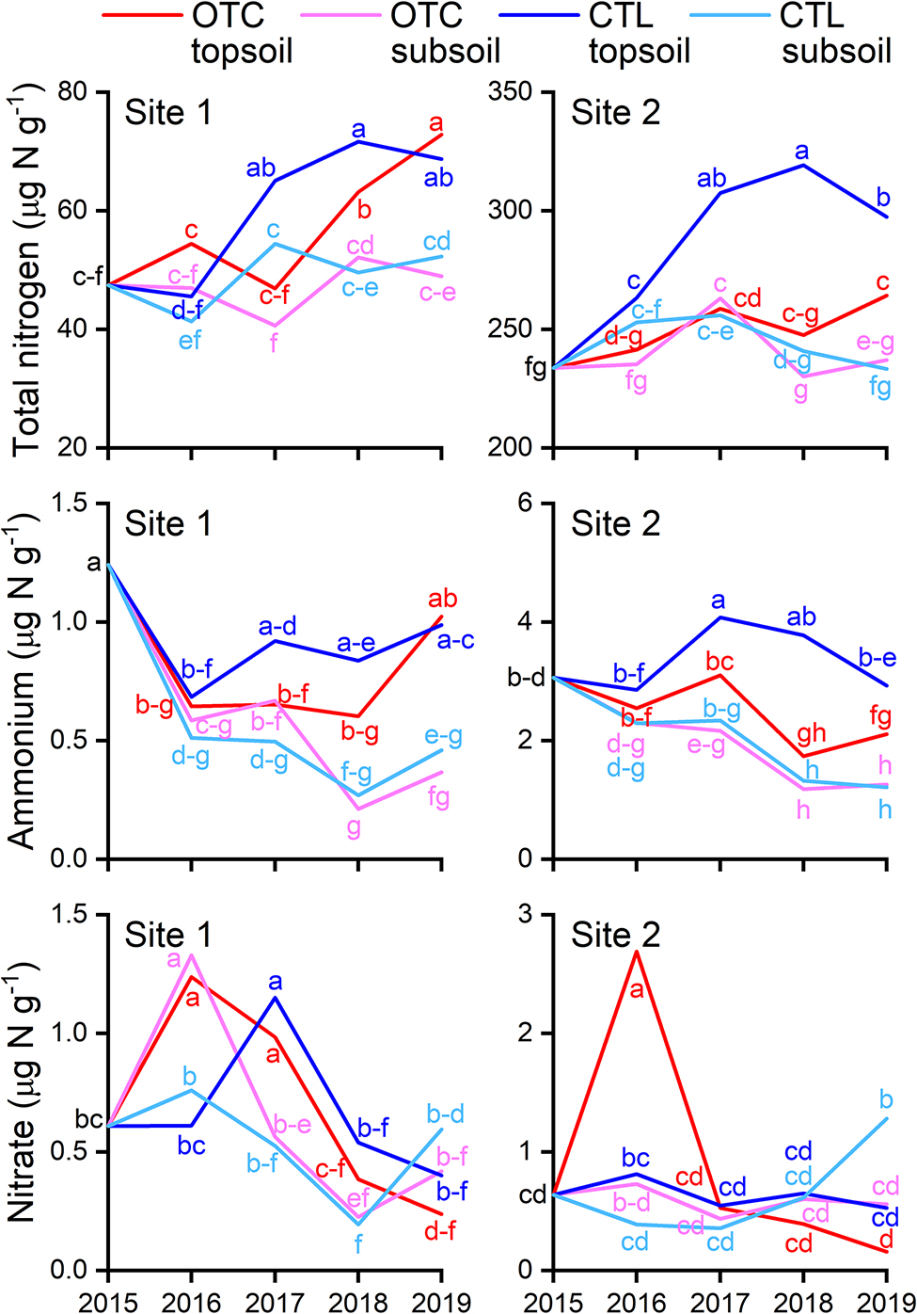
Total nitrogen, ammonium, and nitrate contents of soils at Sites 1 and 2. OTC, open-top chamber plots; CTL, control plots; topsoil, depth of 0–2‍ ‍cm; subsoil, depth of 2–4‍ ‍cm. Different letters denote a significant difference (*P*<0.05). Total nitrogen is expressed as μg N g^–1^ for direct comparisons with ammonium and nitrate contents.

Year-to-year changes in TN, ammonium, and nitrate are shown in Fig. 3 (units: μg N g^–1^ dry soil). TN increased over time in the topsoil at both sites, with a significantly greater rise in the CTL plot than in the OTC plot at Site 2 (*P*<0.05). Ammonium generally decreased over time at both sites (*P*<0.05). Nitrate increased and then returned to its initial levels.

### AOPs

**Fig. 4.**
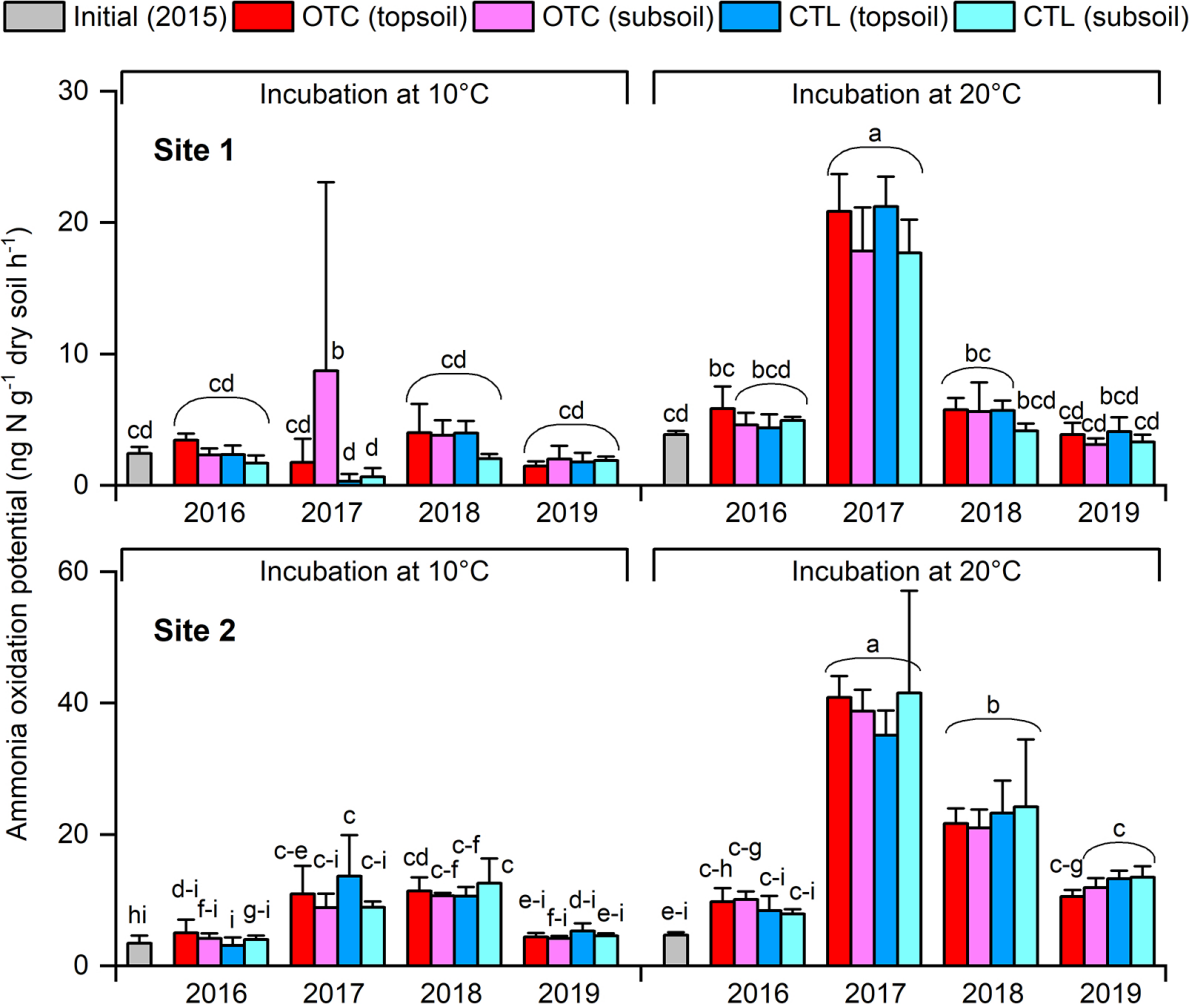
Ammonia oxidation potentials of soils collected from Sites 1 and 2 under incubation temperatures of 10°C and 20°C. OTC, open-top chamber plots; CTL, control plots; topsoil, depth of 0–2‍ ‍cm; subsoil, depth of 2–4‍ ‍cm. Different letters denote a significant difference (*P*<0.05).

Changes in AOPs are shown in Fig. 4. At Site 1, initial AOPs in 2015 were 2.4 and 3.9‍ ‍ng N g^–1^ dry soil h^–1^ at 10°C and 20°C, respectively, with no significant differences between the OTC and CTL plots or soil depths. Although changes in AOPs at 10°C were unclear, they significantly increased at 20°C in 2017, peaking at 19‍ ‍ng N g^–1^ dry soil h^–1^ before returning to initial levels (*P*<0.05). At Site 2, initial AOPs in 2015 were 3.5 and 4.7‍ ‍ng N g^–1^ dry soil h^–1^ at 10°C and 20°C, respectively. At this site, no significant differences were observed between treatments or soil depths. At both incubation temperatures, AOPs increased and then returned to original levels (*P*<0.05); the maximum at 20°C reached 39‍ ‍ng N g^–1^ dry soil h^–1^ in 2017. Comparisons of sites under identical conditions showed that AOPs at Site 2 were three-fold higher on average (range 0.4- to 15-fold) than Site 1. However, the incubation at 10°C for Site 1 in 2017 had five undetectable AOPs out of 12 samples; therefore, that pair was excluded.

### Microbial properties

**Fig. 5.**
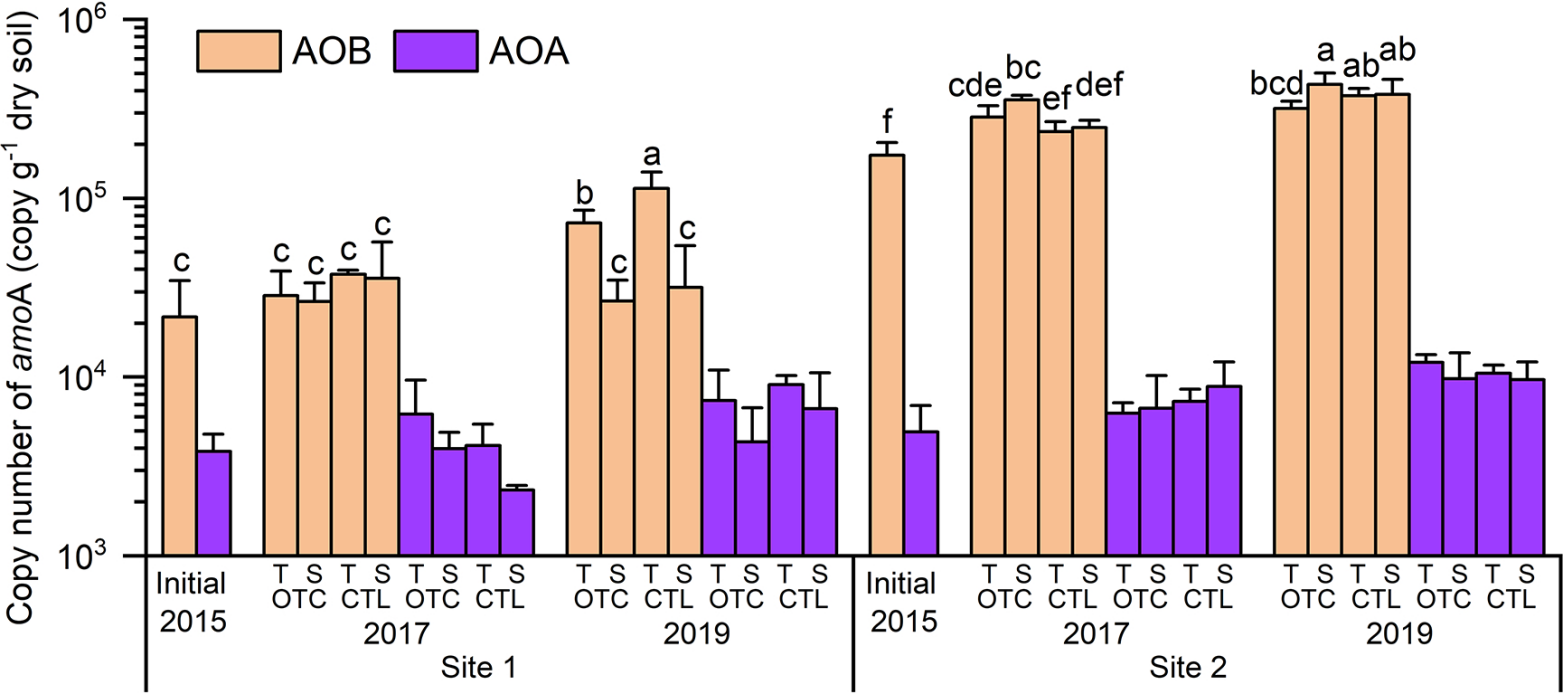
Copy numbers of ammonia-oxidizing bacteria (AOB)-*amo*A and archaea (AOA)-*amo*A. OTC, open-top chamber plot; CTL, control plot; T, topsoil (depth of 0–2‍ ‍cm); S, subsoil (depth of 2–4‍ ‍cm); values in parentheses denote the standard deviation (*n*=3); different letters for AOB denote a significant difference within the site (*P*<0.05).

Comparisons of *amo*A gene copy numbers between sites for the same conditions were performed: in the initial (2015) and each combination of OTC/CTL and topsoil/subsoil (2017 and 2019), AOB copy numbers were 5- to 15-fold higher than AOA at Site 1 and 26- to 28-fold higher at Site 2 (Fig. 5); therefore, anal­yses focused on AOB. Comparisons of sites showed that AOB copy numbers at Site 2 were on average nine-fold (3- to 16-fold) higher than those at Site 1. At Site 1, AOB copy numbers in topsoil significantly increased from 2015 to 2019, with a greater increase in the CTL plot than in the OTC plot (*P*<0.05). At Site 2, AOB copy numbers significantly increased from 2015 to 2019 in all treatments and soil depths (*P*<0.05). No significant OTC effect was observed (Fig. 5).

**Fig. 6.**
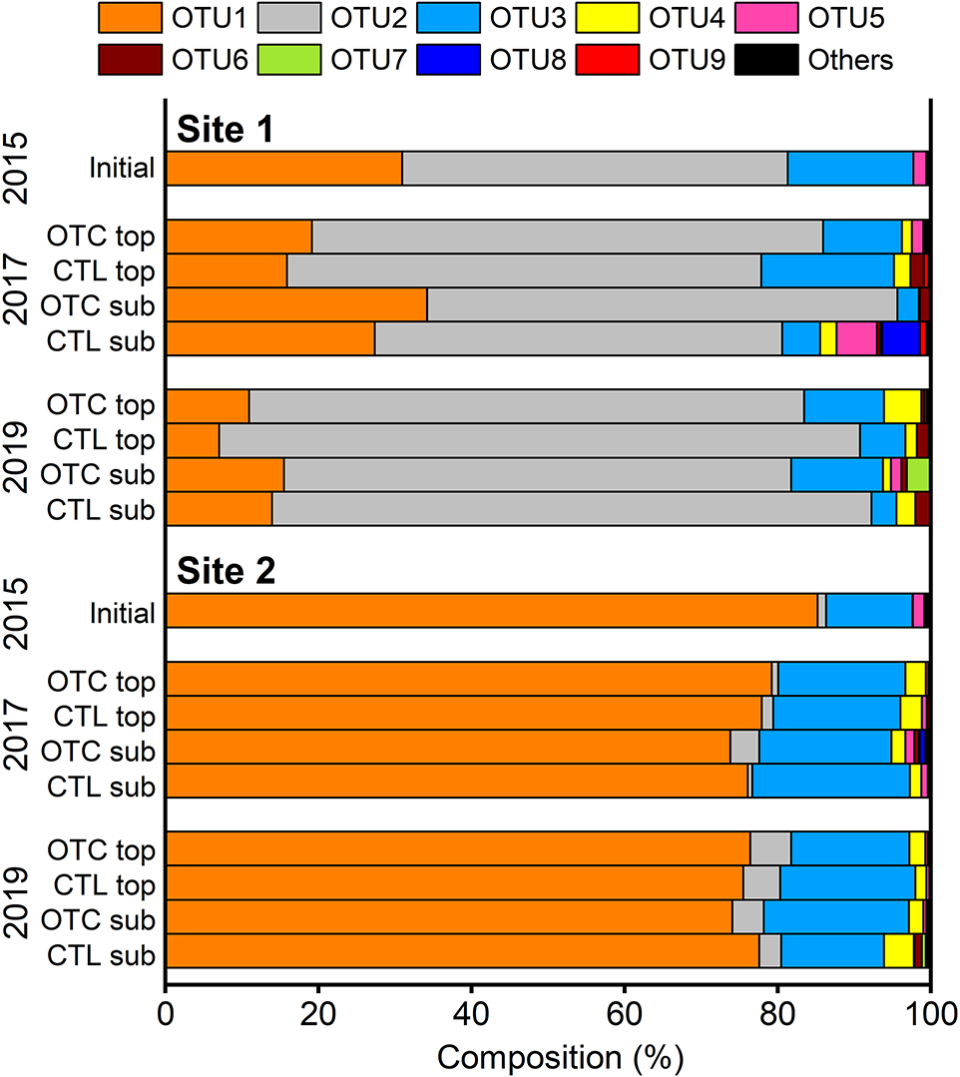
Relative composition of operational taxonomic units (OTUs) of the *amo*A gene of ammonia-oxidizing bacteria (AOB). OTC, open-top chamber plots; CTL, control plots; topsoil, depth of 0–2‍ ‍cm; subsoil, depth of 2–4‍ ‍cm.

The composition of AOB-*amo*A OTUs is shown in Fig. 6. OTU1, OTU2, and OTU3 appeared at both sites under all conditions. OTU1 dominated at Site 2 (mean 77%, range 74–85%). OTU2, which dominated at Site 1 (mean 66%, range 50–84%), was a minor component at Site 2 at which deglaciation occurred earlier. OTU3, although less abun­dant‍ ‍(mean 13%, range 3–21%), was consistently present at both sites. OTU4, nearly absent in 2015, accounted for a small percentage during the experiment (2% on average, excluding initial). The OTU composition of each sample is shown in Table S2 in the Supplementary Materials.

**Fig. 7.**
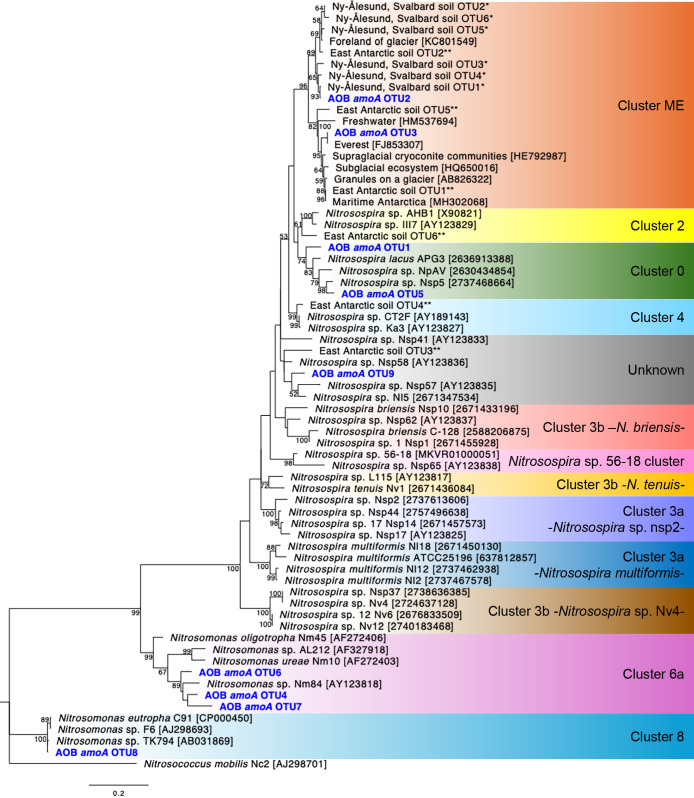
Phylogenetic tree of AOB-*amo*A OTUs (blue bold text) in soils on the foreland of Austre Brøggerbreen, Svalbard. A maximum-likelihood tree was generated based on 413 aligned nucleotide sites of the *amo*A gene. Maximum-likelihood bootstrap values (%) were calculated with 1,000 replicates, and bootstrap values >50 are shown at the tree nodes. Accession numbers in the DNA database (DDBJ/EMBL/GenBank or JGI) are shown in square brackets. We also referred to *amo*A sequence information reported in previous studies, including * OTUs from Ny-Ålesund, Svalbard soil under climax *Salix polaris*–moss vegetation (Hayashi *et al.*, 2016) and ** OTUs from East Antarctic soil (Hayashi *et al.*, 2020).

The phylogenetic tree of AOB-*amo*A OTUs (Fig. 7) shows that OTU2 and OTU3, dominant at Site 1, belong to Cluster ME, while OTU1, dominant at Site 2, belongs to Cluster 0; all belong to *Nitrosospira*. Minor OTUs, OTU5 (Cluster 0) and OTU9 (unknown cluster), were also *Nitrosospira*, whereas OTU4, OTU6, and OTU7 (Cluster 6a) and OTU8 (Cluster 8) were *Nitrosomonas*. Cluster composition ratios showed that Cluster ME and Cluster 0 together accounted for 90.8–99.4% (mean 96.5%) of the total (Fig. S5 in the Supplementary Materials).

## Discussion

### Effects of OTC

The methods and limitations of OTC experiments in Arctic and alpine tundra were extensively reviewed by Hollister *et al.* (2022), drawing on more than 30 years of experience by the International Tundra Experiment network. OTCs are widely used for passive warming due to their low cost, simple installation, and lack of the need for external power, making them practical for remote long-term studies. They typically raise annual mean temperatures by 1–2°C. Although shapes and materials vary, the basic conical hexagonal design, which was used in this study, is standard.

OTCs work mainly by capturing shortwave radiation during the day and reducing wind-driven heat loss; however, nighttime radiative cooling may offset this effect. Their warming effect varies with weather and site conditions and may affect snow depth and snowmelt timing by trapping drifting snow. The warming effect is generally stronger on the surface and weaker in deeper layers. Many studies have found limited direct impacts on soil microbes and decomposition (Hollister *et al.*, 2022).

In the present study, OTCs produced significant warming of 0.39°C at Site 1 and 0.56°C at Site 2 (Table 1), which were lower than typical values. Cold air continuously flowing from upstream glaciers may have cooled the OTCs and reduced warming. Although OTCs often decrease soil moisture in polar semideserts (Hollister *et al.*, 2022), a slight increase in soil moisture was observed (Table 1), which may reflect limited warming. Differences in soil moisture between the OTC and CTL plots showed spikes at the end of the snowmelt period (Fig. S4), indirectly suggesting longer snow cover within OTCs.

### Effects of soil homogenization

Soils were collected in 2015, sieved, mixed, and homogenized to equalize initial conditions, which inevitably disturbed the native microbiota. The observed increase in TN (Fig. 3), temporary rise in AOP (Fig. 4), and emergence of AOB OTU4 after 2015 (Fig. 6) may reflect this disturbance. This is reasonable because the OTC treatment did not significantly change soil properties, AOPs, or the AOB composition (Fig. 3, 4, and 6).

While the homogenization effect is difficult to quantify, it was necessary to reduce site differences that may mask treatment signals. Given the small warming effect, homogenization helped maximize the chance of detecting subtle responses. Chronosequence studies, often used for succession research in glacier forelands (*e.g.*, Hodkinson *et al.*, 2003; Yoshitake *et al.*, 2018; Kim *et al.*, 2022), infer long-term changes efficiently, but are limited by variations in microtopography, soil properties, and microclimate and also by the assumption of identical succession pathways.

### Soil properties on ammonia oxidation

Site 2, exposed for longer since glacier retreat, had higher TN and inorganic N than Site 1 (Fig. 3). The two-way ANOVA showed a limited OTC effect: TN slightly increased at Site 1 (*P*<0.1), while TN and ammonium significantly increased at Site 2 (*P*<0.01), with no other significant changes.

Soils were alkaline (pH 8–9, Table S1), reflecting the carbonate-rich geology (Nowak and Hodson, 2014). Factors affecting nitrifier activity include pH, ammoniacal N concentrations, oxygen, moisture, and temperature, with pH being critical (Nicol *et al.*, 2008; Prosser and Nicol, 2008). AOB generally dominates at pH >5.5–6.0, while AOA prefers acidic soils (Prosser and Nicol, 2008), which is consistent with the present results (Fig. 5).

Ammoniacal N exists in pH-dependent equilibrium: more ammonia is present at pH >7, facilitating volatilization to the atmosphere (Hayashi *et al.*, 2008). Although high pH favors nitrifiers using ammonia (Prosser and Nicol, 2008), greater volatilization may reduce available substrates. Nevertheless, nitrifiers were present in these alkaline soils (Fig. 5) and showed active AOPs (Fig. 4).

### Characteristics of AOPs

Hayashi *et al.* (2016) reported AOPs of 2.3–14.1‍ ‍ng‍ ‍N‍ ‍g^–1^ dry soil h^–1^ at 10°C in mineral soils under climax *Salix polaris*–moss vegetation, with nearby bare soils showing 1.1‍ ‍ng N g^–1^ dry soil h^–1^. In the present study, initial AOPs at 10°C were 2.4 (Site 1) and 3.5 (Site 2) ng‍ ‍N‍ ‍g^–1^ dry soil h^–1^; Site 2 increased to 11.0‍ ‍ng N g^–1^ dry soil h^–1^ in 2017–2018 (Fig. 4), similar to climax levels. In contrast, *in situ* gross nitrification rates at 7°C reported by Wild *et al.* (2013) were markedly higher: 100–110‍ ‍ng N g^–1^ dry soil h^–1^ in heath tundra in Greenland and tussock tundra in northeastern Siberia, and approximately 230‍ ‍ng N g^–1^ dry soil h^–1^ for shrub tundra in northeastern Siberia. At higher temperatures, tundra soils showed higher potential. Canadian Arctic soils reached 21–178‍ ‍ng N g^–1^ dry soil h^–1^ at 20°C with 8‍ ‍mM ammonium (Banerjee and Siciliano, 2012). Alves *et al.* (2013) found rates of 10–200‍ ‍ng N g^–1^ dry soil h^–1^ at 15°C with 1.7–2.5‍ ‍mM ammonium in Svalbard. In the present study, AOPs at 20°C were 3.9 and 4.7‍ ‍ng N g^–1^ dry soil h^–1^ at Sites 1 and 2, respectively, with maximums of 19 and 39‍ ‍ng N g^–1^ dry soil h^–1^ (Fig. 4), which were near the lower end of these ranges. Since our study sites were located on the glacial foreland where weathered substrate, organic matter, and microorganisms had been removed by glacial erosion, lower microbial activity and AOPs than in other tundra sites were reasonable.

Despite mean soil temperatures below freezing point (Table 1), daily mean temperatures often exceeded 10°C, reaching up to 14.0°C at Site 1 and 13.5°C at Site 2 (Fig. S4); noon peaks were 20.2°C at Site 1 and 19.5°C at Site 2 (data not shown). Therefore, 20°C is realistic for local nitrifiers. Hayashi *et al.* (2016) argued that 20°C was too high for active-layer soils under moss on permafrost, but not for exposed forelands.

### Characteristics of AOB diversity

While the OTC treatment did not affect AOB-*amo*A *α*-diversity, clear interannual variations were observed at Site 1, and diversity markedly differed between the two sites (Fig. 6, Table S3 and S4). Based on *amo*A copy numbers, AOB were more abundant than AOA (Fig. 5), and AOB copy numbers correlated with AOPs (Fig. S6). The dominant AOB, in terms of the copy number, were OTU1 to OTU3 (Fig. 6). These results indicate that OTU1 to OTU3 played the dominant role in driving AOPs at study sites.

The phylogenetic anal­ysis showed that OTU1 belonging to Cluster 0 (Fig. 7) was dominant at Site 2, where deglaciation occurred earlier, but was less abundant than OTU2 at Site 1 (Fig. 6). OTU1 has not been detected in mineral soils under climax *Salix polaris*–moss vegetation in Ny-Ålesund, a location near the present study area (Hayashi *et al.*, 2016), suggesting that it represents an AOB lineage unique to this glacier forefield that has become dominant over time. OTU2 and OTU3 belong to Cluster ME (Fig. 7), originally found near Mount Everest (Zhang *et al.*, 2009). OTU2 falls within the same subcluster as six OTUs from Ny-Ålesund climax soils and one OTU from East Antarctic mineral soils, and OTU3 belongs to a different subcluster that includes another East Antarctic OTU (Hayashi *et al.*, 2016, 2020) (Fig. 7). Cluster ME has also been identified in the Tibetan Plateau at elevations of 3,200–4,200‍ ‍m (Zhao *et al.*, 2024). These findings suggest that Cluster ME is broadly distributed across polar and alpine regions. However, how this cluster dispersed globally and why it became a minor component at Site 2, where OTU1 dominated (Fig. 6), remain open questions for further research.

Consistent with previous studies (Hayashi *et al.*, 2016, 2020), the dominance of *Nitrosospira* in our soil samples (Fig. 6 and 7) supports its cold adaptation. In the present study, *Nitrosospira* and *Nitrosomonas* accounted for 97 and 3%, respectively, of the mean composition. Similarly, Zhao *et al.* (2024) found that *Nitrosospira* accounted for the majority (93%) of AOB in Tibetan Plateau soils, although *Nitrosomonas* was also present (7%). Sanders *et al.* (2019) reported that *Nitrosospira* has expanded, particularly in cold environments, including permafrost. Urakawa *et al.* (2015) found that two *Nitrosospira* strains belonging to Cluster 0 were able to grow at 4°C. Collectively, these findings and the present results suggest that cold tolerance is pronounced in *Nitrosospira*, highlighting its ecological importance in polar and alpine regions.

Among the factors tested, site and temperature (OTC/CTL) significantly affected AOB *β*-diversity (*P*<0.01). However, the Venn diagram showed that 57.6% of the effect overlapped, with only 0.13% being temperature-specific and 33.3% being site-specific (Fig. S7). This result indicates that the site effect shaped *β*-diversity more strongly than modest OTC warming.

## Conclusions

The present study used OTCs to test passive warming at two sites on the foreland of Austre Brøggerbreen in Svalbard with different retreat histories. Even in early-stage soils, AOPs were similar to or slightly lower than the values reported for other tundra soils. This result suggests that nitrifiers, as well as N-fixing and organic matter-degrading microbes (Yoshitake *et al.*, 2010, 2018), contribute to early-stage ecosystem development in the glacier foreland. High pH appeared to favor AOB dominance. Nine major AOB-*amo*A OTUs were identified, with three accounting for >90% of the community, two of which belonged to Clus­ter‍ ‍ME. The warming effect was small (0.4–0.6°C), and soil‍ ‍homogenization may have introduced disturbance. Although logistically demanding in polar regions, more active warming experiments may be needed to clarify nitrifier responses. Designing experiments that separate homoge­nization effects and minimize site differences, that is, to address the limitations of the chronosequence approach, will be valuable. Since AOPs reflect potential activity under ample substrate, undisturbed field-based studies are also needed to investigate actual processes. As Arctic warming and glacier retreat continue, a more detailed understanding of soil nitrification and its climate response is essential for predicting soil formation, N cycling, and ecosystem changes at high latitudes.

### Data availability

The sequences described in this study have been deposited in the DNA Data Bank of Japan (BioProject number PRJDB35820, DRR Run number DRR710108-DRR710161).

### Sources of Funding

This study was supported by Grants-in-Aid for Scientific Research (No. 26304018) provided by the Japan Society for the Promotion of Science; the National Institute of Polar Research, Japan (Project Research No. KP309); and the Research Institute for Humanity and Nature, Japan (Project No. RIHN14200156).

### Conflicts of Interest

The authors declare that there are no conflicts of interest.

## Citation

Hayashi, K., Ono, K., Tanabe, Y., Hayatsu, M., Tago, K., Ohbayashi, T., et al. (2025) Ammonia Oxidation Property of Soils on the Glacier Foreland of Austre Brøggerbreen, Svalbard: Response to Open-top Chamber Experiments. *Microbes Environ ***40**: ME25058.

https://doi.org/10.1264/jsme2.ME25058

## Supplementary Material

Supplementary Material

## Figures and Tables

**Table 1.** Soil temperature and moisture during the whole experiment period.

		Mean				Maximum			Minimum	
		OTC	CTL	Difference		OTC	CTL		OTC	CTL
Site 1	Soil temperature (°C)	–0.30	–0.69	0.39 ***		14.0	13.9		–11.6	–12.3
	Soil moisture (v/v)	0.102	0.099	0.003 ***		0.324	0.302		0.039	0.044
Site 2	Soil temperature (°C)	–1.02	–1.58	0.56 ***		13.5	13.4		–14.2	–15.7
	Soil moisture (v/v)	0.096	0.094	0.002 ***		0.374	0.358		0.035	0.033

These values were derived from daily means. Experimental period, 24 July 2015–16 July 2019 (Site 1), 27 July 2015–16 July 2019 (Site 2); OTC, open-top chamber plots; CTL, control plots; Difference, Value (OTC)–Value (CTL); ***, *P*<0.001.
