# Supplementary material for: Ammonia Oxidation Property of Soils on the Glacier Foreland of Austre Brøggerbreen, Svalbard: Response to Open-top Chamber Experiments
Source: Microbes Environ. 2025 Nov 20;40(4):ME25058. doi: 10.1264/jsme2.ME25058 (PMC12727203; doi:10.1264/jsme2.ME25058)
Supplement: Supplementary file 1 — Supplementary Material [file 40_25058_s1.pdf]

Supplementary Materials for

Ammonia oxidation property of soils on the glacier foreland of Austre Brøggerbreen, Svalbard: response to open-top chamber experiments

Kentaro Hayashi[1,*], Keisuke Ono[2], Yukiko Tanabe[3], Masahito Hayatsu[2], Kanako Tago[2,4], Tsubasa Ohbayashi[2], Yong Wang[2,5], Luciano Nobuhiro Aoyagi[2,4], Masaki Uchida[3,6]

[1] Research Institute for Humanity and Nature, National Institutes for the Humanities, Kyoto 603-8047, Japan

[2] Institute for Agro-Environmental Sciences, National Agriculture and Food Research Organization, Tsukuba 305-8604, Japan

[3] National Institute of Polar Research, Tachikawa 190-8518, Japan

[4] School of Veterinary medicine, Kitasato University, Sagamihara 252-0373, Japan

[5] TanBIO Inc., Tsukuba 305-0035, Japan

[6] SOKENDAI (The Graduate University for Advanced Studies), Tachikawa 190-8518, Japan

1. Site information

At both sites, Site 1 and Site 2, the ground surface was mostly bare and had a lot of stones and gravel. There were a few higher plants, such as *Saxifraga oppositifolia*; they were fewer at Site 1. Crusts of organic matter and microbes form on the surface of bare soil (Yoshitake et al., 2010; 2018). The field experiment of this study was conducted in locations not affected by higher plants.

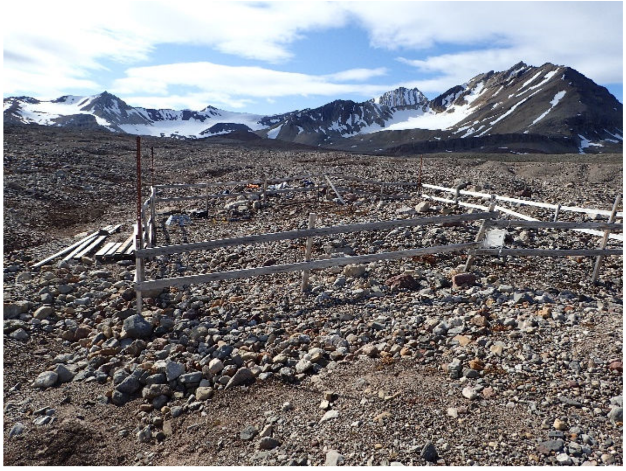

(a) Site 1 view (24 July 2015)

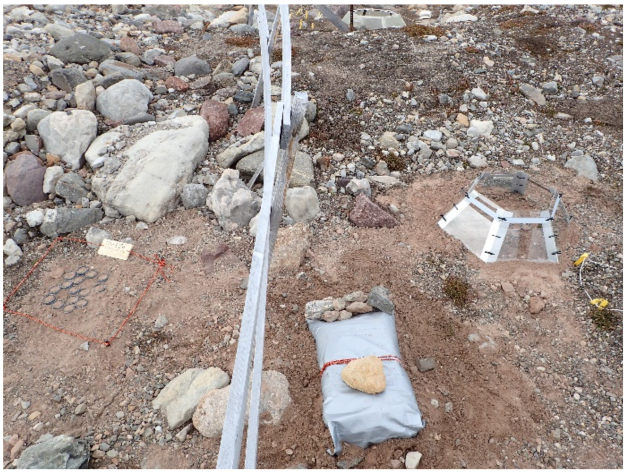

(b) Experimental plots at Site 1 (24 July 2015)

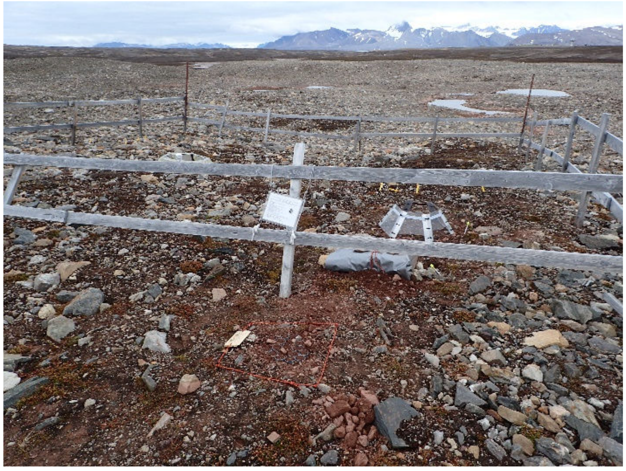

(c) Site 2 view (27 July 2015)

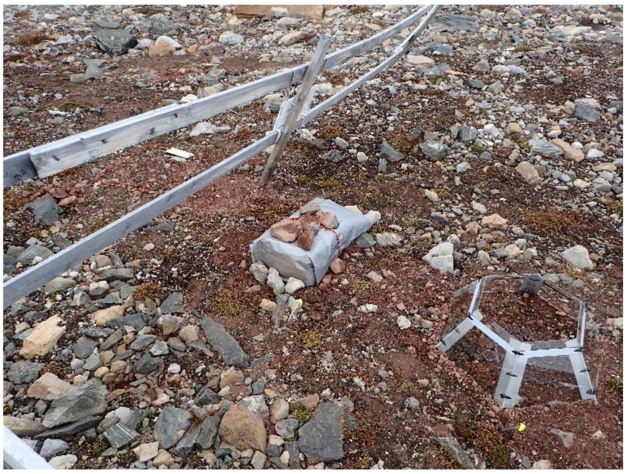

(b) Experimental plots at Site 2 (27 July 2015)

Fig. S1. Site pictures.

2. Experimental setup and soil sampling

To assemble an open-top chamber using six trapezoidal acrylic plates (top 240 mm, bottom 415 mm, height 300 mm, thickness 3 mm per plate), three holes were drilled on each side of each acrylic plate, and the plates were secured with cable ties using stainless steel arms to stably support adjacent acrylic plates and beams to stably support the shape of the hexagonal opening on the top surface (**Fig. S2 (a)**).

The soil cores were made of PVC cylinders (50 mm inner diameter, 60 mm outer diameter, 70 mm height) with a PVC plate with many holes attached to one side as the bottom plate (**Fig. S2 (b)**). Each site had 40 cores. When the experimental plots were set up in 2015, a root-proof permeable sheet cut to the size of the inner diameter was placed in each core, and the core was filled with homogenized initial soil. Prior to this procedure, soil from around the site (approximately 0–20 cm depth) was collected, passed through 4 mm sieve, and well mixed to obtain the homogenized initial soil with a necessary amount enough for at least 40 cores (**Fig. S2 (c) and (d)**).

The cables of sensor (5TM, Decagon) were bundled and buried underground, and the dataloggers (Em50, Decagon; each with five sensor slots) were placed in a waterproof hard case (Pelican, 1500-000-110). Taking into consideration the capacity of the battery and logger, soil temperature and moisture (volumetric water content) data were recorded at 4-hour intervals. After setting up, the hard case was wrapped in insulation material and vinyl sheeting, buried half in the soil, and secured with several stones on top to prevent it from being moved by wind or snowmelt water (**Fig. S2 (e) and (f)**).

Maintenance and soil sampling were carried out once a year in July. At the start of the experiment in 2015, a part of the remaining homogenized soil after making the soil cores was collected as the initial sample. From 2016 onwards, the on-site conditions were checked, the logger data were confirmed and collected, the batteries were renewed, necessary maintenance was performed, and the hard case containing the loggers was reinstalled. Three soil cores per plot were collected for soil and microbial analysis. The collected soil cores were cut on-site into topsoil (0–2 cm depth) and subsoil (approximately 2–4 cm) and placed in plastic bags (**Fig. S3**). Samples were stored at approximately 4°C until transported to the laboratory in Japan. Subsamples for genetic analysis were stored at –80°C until analysis.

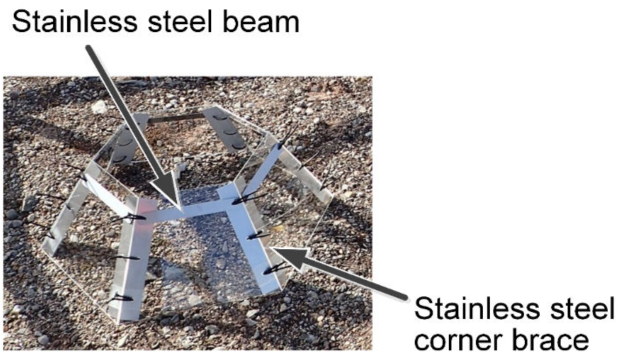

(a) Assembled open-top chamber

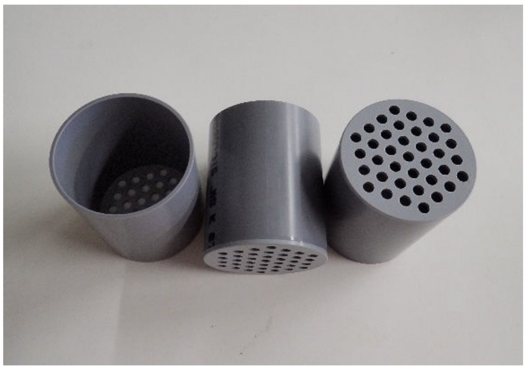

(b) Custom-made polyvinylchloride soil cores

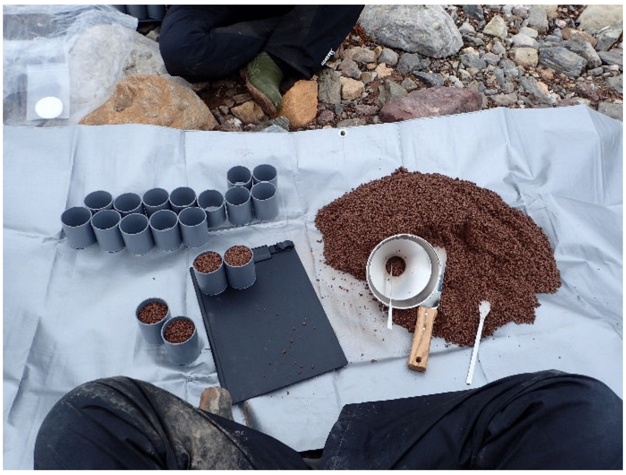

(c) Soil core filling (Site 2, 27 July 2015)

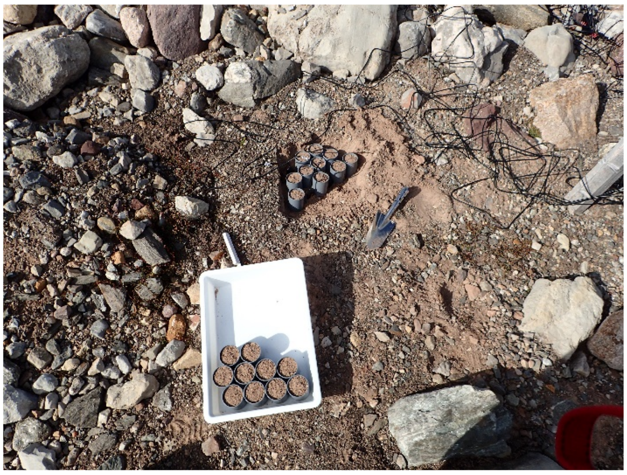

(d) Soil core installation (Site 1, control plot, 24 July 2015)

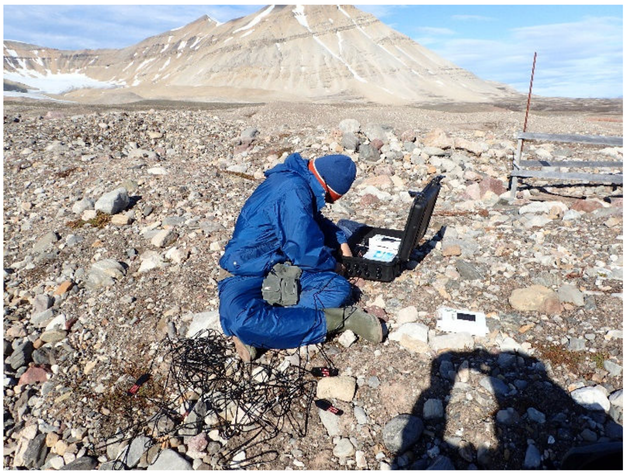

(e) Sensor and datalogger setup (Site 1, 24 July 2015)

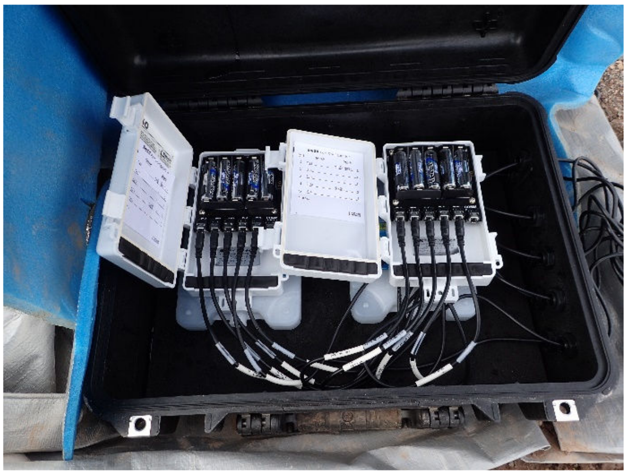

(f) Dataloggers after setup (Site 1, 24 July 2015)

Fig. S2. Preparation for the experiment.

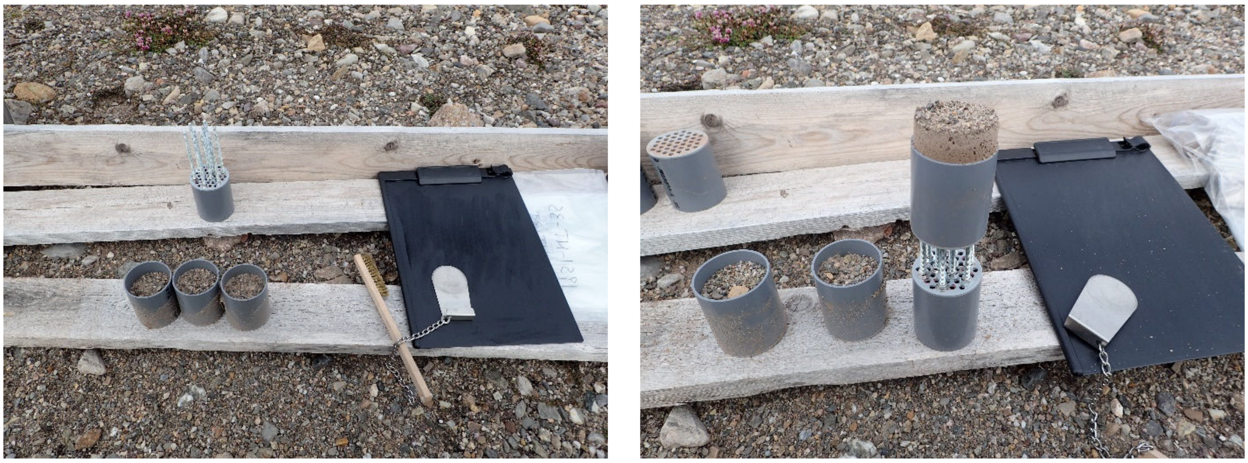

(a) Collected soil cores (Site 1, 17 July 2019) (b) Separation of topsoil and subsoil (Site 1, 17 July 2019)

Fig. S3. Soil core sampling.

3. Microbial analysis

Ammonia oxidation potentials (AOPs), *amo*A gene copy numbers of ammonia-oxidizing bacteria (AOB) and archaea (AOA), operational taxonomic units (OTUs) of AOB-*amo*A with copy numbers higher than AOA, as well as a phylogenetic three including the major AOB-*amo*A OTUs, were analyzed. The AOPs were analyzed for samples collected every summer, similar to the soil samples, while the other items were analyzed for samples collected in the summer of 2015, 2017, and 2019.

AOP measurement

AOPs were determined by measuring nitrite production rates under incubation with sufficient substrate conditions (n = 3). The incubation solution contained 1 mM ammonium sulfate (2 mM ammonium), 10 mM sodium chlorate, and 1 mM monopotassium phosphate buffer (pH 7). Chlorate inhibits nitrite oxidation to nitrate (Belser and Mays 1980); therefore, AOP approximates the potential gross ammonia oxidation rate. Fresh soil with a weight of 2.5 g (2 mm sieved) was added to a 50-mL centrifugation tube, and 10 mL of the incubation solution was added. The tube was shaken well in a tube shaker to create a suspension. Then 1 mL of the suspension was immediately added to a 1.5-mL tube, which was centrifuged at 12,000 rpm for 10 min at 4°C. The supernatant was added to another 1.5-mL tube and stored at 4°C as the sample at zero time. After sampling, the incubation tube was shaken (150 rpm) at 10°C for 24 h. After the shaking incubation, a second supernatant was obtained. The nitrite concentration in the supernatant was determined by using a colorimetric method, and the nitrite production rate per unit weight of dry soil was then calculated as the AOP.

amoA gene copy number

Soil total DNA was extracted by using a FastDNA spin kit for soil (Qbiogene/MP Biomedicals, Solon, OH, USA) (n = 3) without skim milk as described in Morimoto *et al.* (2008). DNA was finally eluted with 80 µL of the DNase/pyrogen-free water (DES) included in the kit. The extracted soil DNA was then purified by using a DNA Clean and Concentrator-25 kit (Zymo Research Corp., Orange, CA, USA). The purified DNA was eluted with 80 µL of DES

and stored at –20°C until analysis.

AOB- and AOA-*amo*A gene copy numbers were quantified by using a SYBR Green I-based real-time PCR technique and the primer sets amoA-1F (5′-GGG GTT TCT ACT GGT GGT-3′) (Rotthauwe *et al.*, 1997)/amoA-2IR (5′-CCC CTC IGI AAA GCC TTC TTC-3′) (Avrahami *et al.*, 2003) for AOB-*amo*A and AOA amoA19IF (5′-ATG GTC TGG CTI AGA CG-3′) (Morimoto *et al.*, 2011)/CrenamoA616r (5′-GCC ATC CAT CTG TAT GTC CA-3′) (Tourna *et al.*, 2008) for AOA-*amo*A. Real-time PCR was performed on a StepOne Plus real-time PCR system (Applied Biosystems, Foster City, CA, USA). Quantitative PCR for AOB-*amo*A was performed under the same conditions as described previously (Shimomura *et al.*, 2012). PCR for AOA-*amo*A was performed in 20-μL reaction mixtures containing 1× SYBR Premix Ex Taq (Takara, Kyoto, Japan), 1 μM of each primer, 0.2 mg mL–1 of bovine serum albumin (BSA), 0.4 μL of ROX Reference Dye (Takara), and about 10 ng of soil DNA (1 μL). Thermal cycling conditions for AOA-*amo*A were 10 min at 95°C, followed by 40 cycles of 30 s at 94°C, 30 s at 55°C, and 1 min at 72°C, and a final elongation step for 10 min at 72°C (Levičnik-Höfferle *et al.*, 2012). To create a standard curve for AOA-*amo*A quantification, tenfold serial dilutions of linearized pGEM-T Easy vector (Promega, Madison, WI, USA) including a soil AOA-*amo*A gene fragment (AB713533) were prepared. The standard curve was generated by plotting the threshold cycle for each standard calculated by StepOne software, ver. 2.1 (Applied Biosystems). The determination coefficients and amplification efficiencies of the standard curves were more than 0.99 and 90%, respectively.

AOB-amoA diversity analysis

A high-throughput sequencing technique was used to analyze AOB-*amo*A gene diversity. The AOA-*amo*A gene was not included in the analysis because its copy numbers were consistently lower than those of AOB. AOB-*amo*A gene was amplified by 25cycles PCR using primers amoA-1F /amoA-2R-GG (Nicolaisen and Ramsing, 2002) with an adaptor sequence. The PCR products were purified with AMPure beads, and after that, an 8-cycle PCR was performed with primers containing unique 8 bp-index sequence to mark samples. The purified products were pooled in equimolar and paired-end sequenced (2 × 300) on an Illumina MiSeq platform (Illumina, SanDiego CA). This *amo*A sequencing were outsourced to Seibutsu Giken Co., Ltd (Kanagawa, Japan).

The FASTQ files corresponding to AOB-*amo*A were processed using an R package DADA2 (version 1.10.1) (Callahan *et al.*, 2016) and Mothur (version 1.42.2) (Schloss *et al.*, 2009). Briefly, all FASTQ files were quality inspected using DADA2. To remove the 3’-end with low quality, all sequences were trimmed to 290 nt in forward and 215 nt in reverse sequence files. Then, sequences were denoised, followed by dereplication of sequences and removal of chimeras using DADA2. The sequences generated from DADA2 were clustered into operational taxonomic units (OTUs) with a cut-off value of 0.05 using Mothur as previously described (Tago *et al.*, 2015). Based on the OTU information, the alpha-diversity indices of AOB were calculated using Mothur. Local BlastN search against reference *amo*A sequences, which were collected from the NCBI database, was performed to decide sequence identity using NCBI Blast+. A multiple sequence alignment of the AOB-*amo*A gene was constructed with MAFFT on the EMBL-EBI server (Katoh *et al.*, 2002). A phylogenetic tree was generated for the AOB-*amo*A gene using the maximum likelihood (ML) method with the removal of gap-including and ambiguous sites and with a bootstrap analysis (1,000 replicates) in MEGA software version 10.1.8 (Kumar *et al.*, 2018; Stecher *et al.*, 2020). We selected the Tamura-Nei model of nucleotide substitutions with gamma distributed and invariant sites (G+I) (Tamura and Nei, 1993).

References for microbial analysis

Avrahami, S., Liesack, W., and Conrad, R. (2003) Effects of temperature and fertilizer on activity and community structure of soil ammonia oxidizers. *Environ Microbiol* **5**: 691–705.

Belser, L.W., and Mays, E.L. (1980) Specific inhibition of nitrite oxidation by chlorate and its use in assessing nitrification in soils and sediments. *Appl Environ Microbiol* **39**: 505–510.

Callahan, B.J., McMurdie, P.J., Rosen, M.J., Han, A.W., Johnson, A.J.A., and Holmes, S.P. (2016) DADA2: High-resolution sample inference from Illumina amplicon data. *Nat Methods* **13**: 581–583.

Katoh, K., Misawa, K., Kuma, K., and Miyata, T. (2002) MAFFT: a novel method for rapid multiple sequence alignment based on fast Fourier transform. *Nucleic Acids Res* **30**: 3059–3066.

Kumar, S., Stecher, G., Li, M., Knayz, C., and Tamura, K. (2018) MEGA X: molecular evolutionary genetics analysis across computing platforms. *Mol Biol Evol* **35**: 1547–1549.

Levičnik-Höfferle, Š, Nicol, G.W., Ausec, L., Mandić-Mulec, I., and Prosser, J.I. (2012) Stimulation of thaumarchaeal ammonia oxidation by ammonia derived from organic nitrogen but not added inorganic nitrogen. *FEMS Microbiol Ecol* **80**: 114–123.

Morimoto, S., Ogawa, N., Hasebe, A., and Fuji, T. (2008) Isolation of effective 3-chlorobenzoate-degraders in soil using community analyses by PCR-DGGE. *Microbes Environ* **23**: 285–292.

Morimoto, S., Hayatsu, M., Hoshino, Y.T., Nagaoka, K., Yamazaki, M., Karasawa, T., Takenaka, M., Akiyama, H. (2011) Quantitative analyses of ammonia-oxidizing archaea (AOA) and ammonia oxidizing bacteria (AOB) in fields with different soil types. *Microbes Environ* **26**: 248–253.

Nicolaisen, M.H., and Ramsing, N.B. (2002) Denaturing gradient gel electrophoresis (DGGE) approaches to study the diversity of ammonia-oxidizing bacteria. *J Microbiol Methods* **50**: 189–203.

Rotthauwe, J.H., Witzel, K.P., and Liesack, W. (1997) The ammonia monooxygenase structural gene *amo*A as a functional marker: molecular fine-scale analysis of natural ammonia-oxidizing populations. *Appl Environ Microbiol* **63**: 4704–4712.

Schloss, P.D., Westcott, S.L., Ryabin, T., Hall, J.R., Hartmann, M., Hollister, E.B., *et al.* (2009) Introducing mothur: open-source, platform-independent, community-supported software for describing and comparing microbial communities. *Appl Environ Microbiol* **75**: 7537–7541.

Shimomura, Y., Morimoto, S., Hoshino, Y.T., Uchida, Y., Akiyama, H., and Hayatsu, M. (2012) Comparison among *amo*A primers suited for quantification and diversity analyses of ammonia-oxidizing bacteria in soil. *Microbes Environ* **27**: 94–98.

Stecher, G., Tamura, K., and Kumar, S. (2020) Molecular Evolutionary Genetics Analysis (MEGA) for macOS. *Mol. Biol. Evol* **37**: 1237–1239.

Tago, K., Okubo, T., Shimomura, Y., Kikuchi, Y., Hori, T., Nagayama, A., and Hayatsu, M. (2015) Environmental factors shaping the community structure of ammonia-oxidizing bacteria and archaea in sugarcane field soil. *Microbes Environ* **30**: 21–28.

Tamura, K., and Nei, M. (1993) Estimation of the number of nucleotide substitutions in the control region of mitochondrial DNA in humans and chimpanzees. *Mol Biol Evol* **10**: 512–526.

Tourna, M., Freitag, T.E., Nicol, G.W., and Prosser, J.I. (2008) Growth, activity and temperature responses of ammonia-oxidizing archaea and bacteria in soil microcosms. *Environ Microbiol* **10**: 1357–1364.

4. Supplementary figures and tables of the study results

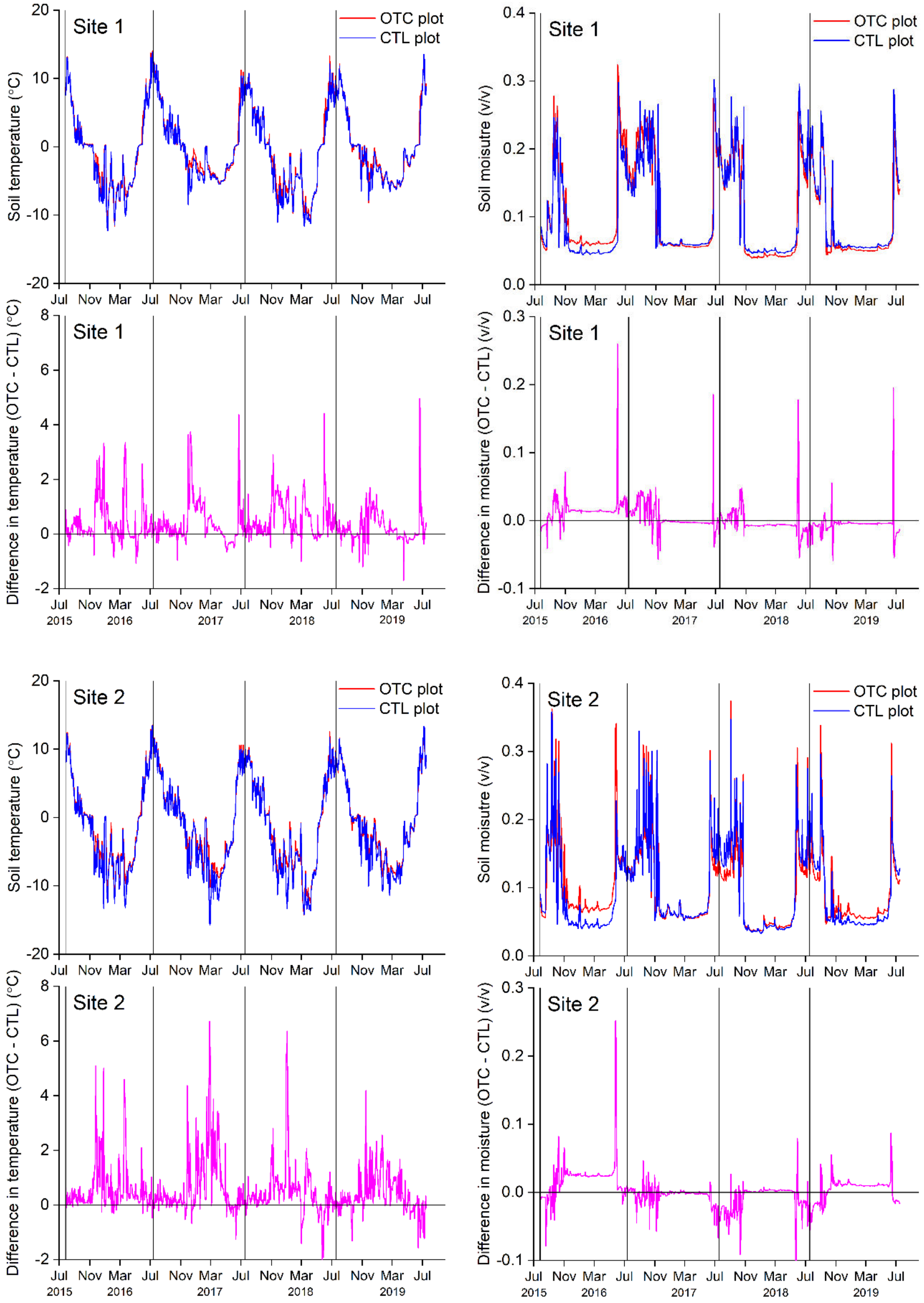

Fig. S4. Soil temperature and moisture (volumetric water content) during the research period.

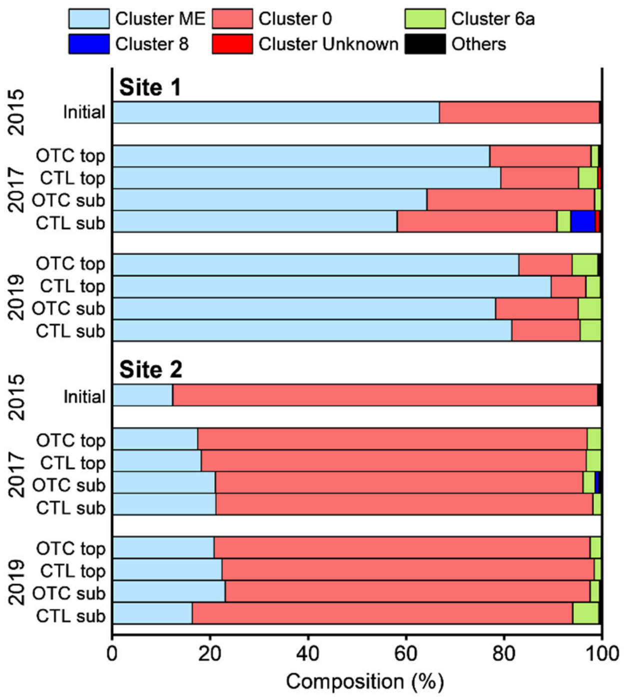

Fig. S5. Relative abundance of the clusters of *amo*A gene diversity of ammonia oxidizing bacteria (AOB) in soils on glacier foreland of Austre Brøggerbreen, Svalbard. AOB-*amo*A subclades were identified based on phylogenetic divergence (**Fig. 7**, main text).

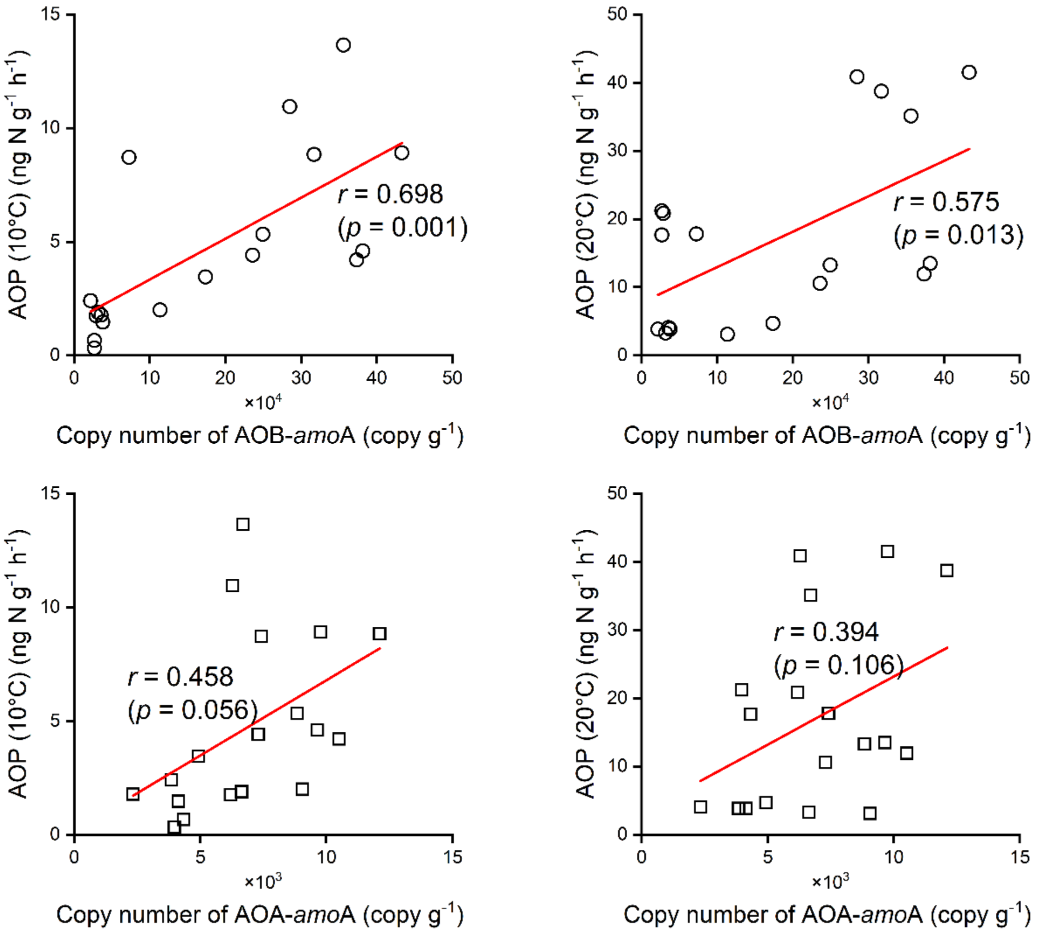

Fig. S6. Relationship between ammonia oxidation potential (AOP) and copy numbers of ammonia-oxidizing bacteria (AOB) and archaea (AOA) *amoA* gene.

PERMANOVA test (Two-way ANOVA)		
	p	
Site	0.0010	Site 1, 2
Temperature	0.0010	OTC, CTL
Year	0.3926	2017, 2019
Layer	0.6284	topsoil, subsoil

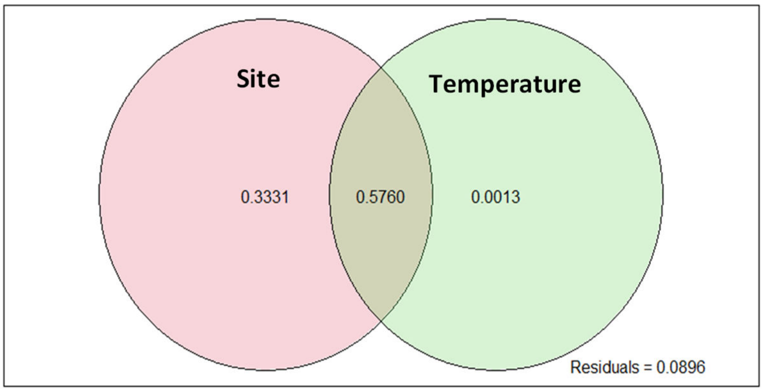

Fig. S7. Effects of environmental factors on the β-diversity of ammonia oxidizing bacteria (AOB).

Table S1. Soil properties.

Site	Treat-ment	Layer	Year	Soil pH (H_2O)		Total carbon (%)		Total nitrogen (%)		CN ratio		Ammonium (μg N g^{-1} soil)		Nitrate (μg N g^{-1} soil)		Nitrite (μg N g^{-1} soil)	
				Mean	SD	Mean	SD	Mean	SD	Mean	SD	Mean	SD	Mean	SD	Mean	SD
Site 1	Initial	Total	2015	9.4	0.2	0.115	0.025	0.0047	0.0006	24.0	2.3	1.242	0.659	0.609	0.438	0.004	0.000
Site 1	OTC	Topsoil	2016	9.5	0.3	0.118	0.014	0.0054	0.0002	21.7	2.7	0.645	0.066	1.237	0.386	0.003	0.001
Site 1	OTC	Topsoil	2017	9.6	0.1	0.096	0.014	0.0047	0.0003	20.5	3.2	0.653	0.246	0.985	0.311	0.006	0.004
Site 1	OTC	Topsoil	2018	9.2	0.3	0.103	0.006	0.0063	0.0005	16.4	1.5	0.603	0.076	0.384	0.154	0.001	0.000
Site 1	OTC	Topsoil	2019	9.2	0.1	0.126	0.006	0.0073	0.0007	17.4	2.5	1.024	0.086	0.238	0.007	0.000	0.000
Site 1	OTC	Subsoil	2016	9.5	0.2	0.115	0.004	0.0047	0.0003	24.5	1.9	0.586	0.023	1.329	0.052	0.009	0.002
Site 1	OTC	Subsoil	2017	9.5	0.2	0.104	0.006	0.0041	0.0003	25.5	1.6	0.669	0.034	0.563	0.123	0.010	0.004
Site 1	OTC	Subsoil	2018	9.1	0.4	0.109	0.027	0.0052	0.0004	20.9	4.0	0.212	0.028	0.226	0.025	0.002	0.001
Site 1	OTC	Subsoil	2019	8.9	0.2	0.103	0.012	0.0049	0.0004	21.0	0.6	0.367	0.131	0.418	0.017	0.001	0.001
Site 1	CTL	Topsoil	2016	9.6	0.2	0.101	0.016	0.0046	0.0006	22.1	0.7	0.685	0.060	0.610	0.154	0.004	0.002
Site 1	CTL	Topsoil	2017	9.7	0.0	0.112	0.011	0.0065	0.0006	17.3	0.2	0.921	0.054	1.151	0.284	0.006	0.001
Site 1	CTL	Topsoil	2018	8.9	0.3	0.157	0.003	0.0072	0.0003	21.9	1.2	0.837	0.396	0.539	0.230	0.001	0.001
Site 1	CTL	Topsoil	2019	9.1	0.2	0.119	0.002	0.0069	0.0005	17.4	1.2	0.987	0.053	0.402	0.051	0.001	0.000
Site 1	CTL	Subsoil	2016	9.8	0.1	0.096	0.013	0.0041	0.0005	23.1	1.2	0.513	0.057	0.760	0.020	0.006	0.002
Site 1	CTL	Subsoil	2017	9.5	0.2	0.125	0.034	0.0054	0.0006	22.8	4.1	0.496	0.079	0.525	0.016	0.007	0.000
Site 1	CTL	Subsoil	2018	9.1	0.1	0.115	0.015	0.0050	0.0003	23.1	2.1	0.269	0.062	0.194	0.014	0.001	0.000
Site 1	CTL	Subsoil	2019	9.0	0.4	0.120	0.018	0.0052	0.0006	22.9	0.9	0.460	0.124	0.596	0.045	0.002	0.000
Site 2	Initial	Total	2015	8.8	0.3	0.326	0.027	0.0234	0.0009	13.9	0.6	3.060	0.718	0.641	0.533	0.018	0.003
Site 2	OTC	Topsoil	2016	9.1	0.2	0.329	0.044	0.0241	0.0023	13.6	0.6	2.550	0.190	2.692	0.573	0.072	0.010
Site 2	OTC	Topsoil	2017	8.8	0.2	0.362	0.013	0.0259	0.0009	14.0	0.2	3.102	0.652	0.526	0.618	0.031	0.006
Site 2	OTC	Topsoil	2018	8.6	0.3	0.327	0.030	0.0248	0.0006	13.2	0.9	1.739	0.311	0.393	0.011	0.014	0.002
Site 2	OTC	Topsoil	2019	8.8	0.3	0.347	0.023	0.0264	0.0009	13.1	0.4	2.115	0.255	0.157	0.012	0.002	0.000
Site 2	OTC	Subsoil	2016	9.3	0.2	0.339	0.031	0.0235	0.0004	14.4	1.1	2.299	0.247	0.731	0.123	0.040	0.005
Site 2	OTC	Subsoil	2017	9.0	0.1	0.366	0.023	0.0263	0.0008	13.9	0.6	2.169	0.223	0.436	0.151	0.038	0.004
Site 2	OTC	Subsoil	2018	8.5	0.1	0.319	0.027	0.0230	0.0007	13.9	0.8	1.186	0.152	0.603	0.106	0.011	0.001
Site 2	OTC	Subsoil	2019	8.7	0.3	0.322	0.024	0.0237	0.0010	13.6	0.9	1.260	0.148	0.559	0.118	0.005	0.001
Site 2	CTL	Topsoil	2016	9.1	0.4	0.373	0.032	0.0263	0.0009	14.2	1.5	2.854	0.565	0.814	0.227	0.038	0.002
Site 2	CTL	Topsoil	2017	8.7	0.1	0.410	0.015	0.0308	0.0019	13.3	0.3	4.080	0.665	0.548	0.563	0.052	0.017
Site 2	CTL	Topsoil	2018	8.7	0.4	0.423	0.057	0.0319	0.0024	13.2	0.8	3.778	0.987	0.651	0.799	0.012	0.005
Site 2	CTL	Topsoil	2019	8.9	0.2	0.388	0.018	0.0297	0.0010	13.0	0.2	2.929	0.438	0.527	0.047	0.003	0.000
Site 2	CTL	Subsoil	2016	9.2	0.3	0.356	0.042	0.0253	0.0015	14.0	0.8	2.292	0.101	0.390	0.098	0.029	0.003
Site 2	CTL	Subsoil	2017	8.8	0.4	0.341	0.004	0.0256	0.0005	13.3	0.2	2.344	0.169	0.358	0.054	0.034	0.004
Site 2	CTL	Subsoil	2018	8.4	0.2	0.335	0.042	0.0241	0.0009	13.9	1.2	1.322	0.057	0.614	0.072	0.014	0.009
Site 2	CTL	Subsoil	2019	8.8	0.3	0.302	0.005	0.0233	0.0001	13.0	0.2	1.211	0.199	1.281	0.047	0.005	0.002

Table S2. Relative composition of operational taxonomic units (OTUs) of *amo*A gene of ammonia-oxidizing bacteria (AOB) for each sample.

Site	Year	Sample	Treatment	Soil layer	No. of sequences for analysis	Relative composition of OTUs (%)									
						OTU1	OTU2	OTU3	OTU4	OTU5	OTU6	OTU7	OTU8	OTU9	Others
Site 1	2015	1	Initial	Total	12402	13.9	63.2	23.0	0.0	0.0	0.0	0.0	0.0	0.0	0.0
Site 1	2015	2	Initial	Total	12402	29.3	38.3	26.3	0.0	5.1	0.7	0.0	0.0	0.0	0.3
Site 1	2015	3	Initial	Total	12402	49.8	49.6	0.0	0.0	0.0	0.0	0.0	0.0	0.0	0.7
Site 1	2017	1	OTC	Topsoil	12402	23.1	52.2	21.8	0.2	0.0	0.0	0.5	0.0	0.0	2.3
Site 1	2017	2	OTC	Topsoil	12402	13.8	72.4	9.0	0.1	4.5	0.1	0.0	0.0	0.0	0.0
Site 1	2017	3	OTC	Topsoil	12402	20.5	75.9	0.0	3.6	0.0	0.1	0.0	0.0	0.0	0.0
Site 1	2017	1	OTC	Subsoil	12402	49.6	50.2	0.0	0.0	0.0	0.0	0.0	0.0	0.0	0.2
Site 1	2017	2	OTC	Subsoil	12402	14.9	76.4	8.3	0.3	0.0	0.0	0.0	0.0	0.0	0.0
Site 1	2017	3	OTC	Subsoil	12402	38.1	57.6	0.2	0.0	0.0	4.1	0.0	0.0	0.0	0.0
Site 1	2017	1	CTL	Topsoil	12402	15.4	62.9	13.3	3.1	0.0	5.2	0.0	0.0	0.0	0.0
Site 1	2017	2	CTL	Topsoil	12402	14.2	70.0	11.1	1.9	0.0	0.0	0.0	0.0	2.0	0.8
Site 1	2017	3	CTL	Topsoil	12402	18.1	52.8	27.7	1.4	0.0	0.0	0.0	0.0	0.0	0.0
Site 1	2017	1	CTL	Subsoil	12402	19.3	46.3	3.9	3.2	8.4	1.1	0.5	14.9	2.5	0.0
Site 1	2017	2	CTL	Subsoil	12402	33.2	57.1	8.1	0.7	0.0	0.3	0.0	0.0	0.0	0.7
Site 1	2017	3	CTL	Subsoil	12402	29.6	56.3	2.8	2.6	7.5	0.0	0.1	0.0	0.0	1.1
Site 1	2019	1	OTC	Topsoil	12402	9.0	73.9	11.5	3.2	0.0	0.0	0.0	0.0	1.0	1.5
Site 1	2019	2	OTC	Topsoil	12402	7.3	77.0	8.0	7.7	0.0	0.0	0.0	0.0	0.0	0.0
Site 1	2019	3	OTC	Topsoil	12402	16.4	66.8	11.8	3.7	0.0	1.2	0.0	0.0	0.0	0.0
Site 1	2019	1	OTC	Subsoil	12402	19.8	72.5	7.3	0.4	0.0	0.0	0.0	0.0	0.0	0.0
Site 1	2019	2	OTC	Subsoil	12402	11.1	60.0	21.6	0.9	4.1	2.2	0.0	0.0	0.0	0.1
Site 1	2019	3	OTC	Subsoil	12402	15.7	66.2	7.2	1.7	0.0	0.0	9.3	0.0	0.0	0.0
Site 1	2019	1	CTL	Topsoil	12402	6.4	84.2	4.5	0.8	0.0	3.4	0.0	0.0	0.8	0.0
Site 1	2019	2	CTL	Topsoil	12402	13.2	72.3	9.7	3.7	0.0	1.1	0.0	0.0	0.0	0.0
Site 1	2019	3	CTL	Topsoil	12402	1.5	94.7	3.5	0.0	0.0	0.0	0.0	0.0	0.3	0.0
Site 1	2019	1	CTL	Subsoil	12402	11.4	81.7	0.0	1.4	0.0	5.5	0.0	0.0	0.0	0.0
Site 1	2019	2	CTL	Subsoil	12402	16.2	67.5	9.7	6.0	0.0	0.6	0.0	0.0	0.0	0.0
Site 1	2019	3	CTL	Subsoil	12402	14.1	85.8	0.0	0.1	0.0	0.0	0.0	0.0	0.0	0.0
Site 2	2015	1	Initial	Total	12402	83.8	0.9	12.7	0.0	2.6	0.0	0.0	0.0	0.0	0.0
Site 2	2015	2	Initial	Total	12402	85.5	1.5	8.6	0.2	1.7	0.0	0.0	0.0	0.0	2.5
Site 2	2015	3	Initial	Total	12402	86.3	0.8	12.6	0.0	0.3	0.0	0.0	0.0	0.0	0.0
Site 2	2017	1	OTC	Topsoil	12402	78.9	2.1	17.4	1.2	0.0	0.3	0.0	0.0	0.0	0.0
Site 2	2017	2	OTC	Topsoil	12402	82.1	0.0	15.0	1.9	0.4	0.5	0.0	0.0	0.0	0.1
Site 2	2017	3	OTC	Topsoil	12402	76.7	0.4	17.3	4.9	0.5	0.1	0.0	0.0	0.0	0.0
Site 2	2017	1	OTC	Subsoil	12402	71.5	4.2	18.1	1.4	1.2	1.1	0.0	2.2	0.0	0.4
Site 2	2017	2	OTC	Subsoil	12402	70.7	5.7	20.1	1.6	1.0	0.3	0.2	0.0	0.0	0.2
Site 2	2017	3	OTC	Subsoil	12402	79.2	1.4	13.6	2.4	1.5	0.2	0.1	0.0	0.0	1.5
Site 2	2017	1	CTL	Topsoil	12402	70.1	1.8	22.8	3.2	0.9	0.6	0.0	0.6	0.0	0.0
Site 2	2017	2	CTL	Topsoil	12402	83.7	0.0	13.0	3.0	0.0	0.3	0.0	0.0	0.0	0.0
Site 2	2017	3	CTL	Topsoil	12402	79.9	2.7	14.2	2.1	1.1	0.0	0.0	0.0	0.0	0.0
Site 2	2017	1	CTL	Subsoil	12402	73.8	0.4	23.2	1.9	0.3	0.3	0.0	0.0	0.0	0.0
Site 2	2017	2	CTL	Subsoil	12402	76.2	1.0	19.7	2.3	0.0	0.2	0.6	0.0	0.0	0.0
Site 2	2017	3	CTL	Subsoil	12402	78.3	0.2	18.9	0.3	2.2	0.0	0.0	0.0	0.0	0.0
Site 2	2019	1	OTC	Topsoil	12402	71.4	7.5	17.4	3.2	0.0	0.5	0.0	0.0	0.0	0.1
Site 2	2019	2	OTC	Topsoil	12402	82.1	1.7	15.4	0.0	0.7	0.0	0.0	0.0	0.0	0.0
Site 2	2019	3	OTC	Topsoil	12402	75.7	6.9	13.5	3.1	0.3	0.0	0.0	0.0	0.0	0.5
Site 2	2019	1	OTC	Subsoil	12402	74.0	3.4	20.5	2.0	0.1	0.0	0.0	0.0	0.0	0.0
Site 2	2019	2	OTC	Subsoil	12402	73.2	5.6	18.5	1.4	0.6	0.1	0.0	0.0	0.0	0.7
Site 2	2019	3	OTC	Subsoil	12402	75.0	3.2	18.0	2.3	0.6	0.2	0.0	0.0	0.0	0.8
Site 2	2019	1	CTL	Topsoil	12402	63.8	5.5	28.1	1.8	0.5	0.1	0.0	0.0	0.0	0.1
Site 2	2019	2	CTL	Topsoil	12402	80.2	3.0	14.1	2.3	0.2	0.2	0.0	0.0	0.0	0.0
Site 2	2019	3	CTL	Topsoil	12402	82.6	5.9	10.6	0.2	0.6	0.1	0.0	0.0	0.0	0.0
Site 2	2019	1	CTL	Subsoil	12402	77.8	6.1	11.3	3.8	0.0	0.6	0.1	0.0	0.0	0.1
Site 2	2019	2	CTL	Subsoil	12402	77.9	1.2	15.9	3.2	0.3	0.1	1.3	0.0	0.0	0.2
Site 2	2019	3	CTL	Subsoil	12402	77.1	1.4	13.1	4.6	0.0	2.1	0.0	0.0	0.0	1.8

OTC, open-top chamber plots; CTL, control plots.

Table S3. Alpha-diversity of ammonia oxidizing bacteria.

Site	Year	Sample	Treatment	Soil layer	No. of sequences for analysis	Good's library coverage	No. of OTUs observed (OTU richness)	No. of OTUs estimated (Chao1 richness)	Inverse Simpson index (1/λ)	Shannon's diversity index (H')	Shannon's species evenness (E)
Site 1	2015	1	Initial	Total	12402	100.0%	4	4	2.12	0.90	0.65
Site 1	2015	2	Initial	Total	12402	100.0%	6	6	3.29	1.28	0.72
Site 1	2015	3	Initial	Total	12402	100.0%	3	3	2.03	0.73	0.66
Site 1	2017	1	OTC	Topsoil	12402	100.0%	6	6	2.68	1.13	0.63
Site 1	2017	2	OTC	Topsoil	12402	100.0%	6	6	1.81	0.88	0.49
Site 1	2017	3	OTC	Topsoil	12402	100.0%	4	4	1.62	0.66	0.47
Site 1	2017	1	OTC	Subsoil	12402	100.0%	3	3	2.01	0.70	0.64
Site 1	2017	2	OTC	Subsoil	12402	100.0%	4	4	1.63	0.71	0.52
Site 1	2017	3	OTC	Subsoil	12402	100.0%	4	4	2.09	0.83	0.60
Site 1	2017	1	CTL	Topsoil	12402	100.0%	5	5	2.27	1.11	0.69
Site 1	2017	2	CTL	Topsoil	12402	100.0%	7	7	1.91	0.97	0.50
Site 1	2017	3	CTL	Topsoil	12402	100.0%	4	4	2.57	1.06	0.77
Site 1	2017	1	CTL	Subsoil	12402	100.0%	10	10	3.52	1.57	0.68
Site 1	2017	2	CTL	Subsoil	12402	100.0%	6	6	2.26	0.98	0.54
Site 1	2017	3	CTL	Subsoil	12402	100.0%	7	7	2.43	1.13	0.58
Site 1	2019	1	OTC	Topsoil	12402	100.0%	7	7	1.76	0.92	0.47
Site 1	2019	2	OTC	Topsoil	12402	100.0%	4	4	1.64	0.79	0.57
Site 1	2019	3	OTC	Topsoil	12402	100.0%	6	6	2.04	1.00	0.56
Site 1	2019	1	OTC	Subsoil	12402	100.0%	4	4	1.76	0.77	0.55
Site 1	2019	2	OTC	Subsoil	12402	100.0%	7	7	2.37	1.15	0.59
Site 1	2019	3	OTC	Subsoil	12402	100.0%	5	5	2.10	1.04	0.65
Site 1	2019	1	CTL	Topsoil	12402	100.0%	6	6	1.40	0.65	0.36
Site 1	2019	2	CTL	Topsoil	12402	100.0%	5	5	1.81	0.90	0.56
Site 1	2019	3	CTL	Topsoil	12402	100.0%	4	4	1.11	0.25	0.18
Site 1	2019	1	CTL	Subsoil	12402	100.0%	4	4	1.46	0.63	0.45
Site 1	2019	2	CTL	Subsoil	12402	100.0%	5	5	2.02	0.98	0.61
Site 1	2019	3	CTL	Subsoil	12402	100.0%	3	3	1.32	0.42	0.38
Site 2	2015	1	Initial	Total	12402	100.0%	5	5	1.39	0.55	0.34
Site 2	2015	2	Initial	Total	12402	100.0%	6	6	1.35	0.58	0.32
Site 2	2015	3	Initial	Total	12402	100.0%	4	4	1.31	0.44	0.32
Site 2	2017	1	OTC	Topsoil	12402	100.0%	6	6	1.53	0.65	0.36
Site 2	2017	2	OTC	Topsoil	12402	100.0%	6	6	1.44	0.58	0.32
Site 2	2017	3	OTC	Topsoil	12402	100.0%	6	6	1.61	0.71	0.40
Site 2	2017	1	OTC	Subsoil	12402	100.0%	11	11	1.83	0.95	0.40
Site 2	2017	2	OTC	Subsoil	12402	100.0%	9	9	1.84	0.89	0.41
Site 2	2017	3	OTC	Subsoil	12402	100.0%	10	10	1.55	0.77	0.33
Site 2	2017	1	CTL	Topsoil	12402	100.0%	8	8	1.84	0.88	0.42
Site 2	2017	2	CTL	Topsoil	12402	100.0%	4	4	1.39	0.54	0.39
Site 2	2017	3	CTL	Topsoil	12402	100.0%	5	5	1.52	0.69	0.43
Site 2	2017	1	CTL	Subsoil	12402	100.0%	6	6	1.67	0.70	0.39
Site 2	2017	2	CTL	Subsoil	12402	100.0%	7	7	1.61	0.71	0.36
Site 2	2017	3	CTL	Subsoil	12402	100.0%	6	6	1.54	0.63	0.35
Site 2	2019	1	OTC	Topsoil	12402	100.0%	6	6	1.83	0.88	0.49
Site 2	2019	2	OTC	Topsoil	12402	100.0%	4	4	1.43	0.56	0.40
Site 2	2019	3	OTC	Topsoil	12402	100.0%	7	7	1.67	0.82	0.42
Site 2	2019	1	OTC	Subsoil	12402	100.0%	5	5	1.69	0.75	0.46
Site 2	2019	2	OTC	Subsoil	12402	100.0%	8	8	1.75	0.84	0.40
Site 2	2019	3	OTC	Subsoil	12402	100.0%	9	9	1.68	0.80	0.36
Site 2	2019	1	CTL	Topsoil	12402	100.0%	7	7	2.04	0.92	0.47
Site 2	2019	2	CTL	Topsoil	12402	100.0%	6	6	1.51	0.67	0.37
Site 2	2019	3	CTL	Topsoil	12402	100.0%	6	6	1.44	0.61	0.34
Site 2	2019	1	CTL	Subsoil	12402	100.0%	7	7	1.60	0.79	0.40
Site 2	2019	2	CTL	Subsoil	12402	100.0%	9	9	1.58	0.74	0.34
Site 2	2019	3	CTL	Subsoil	12402	100.0%	6	6	1.63	0.82	0.46

OTC, open-top chamber plots; CTL, control plots.

Table S4. Statistical analysis of α-diversity of ammonia oxidizing bacteria.

Groups	Statistical analysis	Comparison	No. of OTUs observed (OTU richness)	No. of OTUs estimated (Chao1 richness)	Inverse Simpson index (1/λ)	Shannon's diversity index (H')	Shannon's species evenness (E)
Initial 2015	*t*-test *p*	Site 1 vs Site 2	0.1835	0.1835	0.1079	0.0731	0.0044 **
OTC 2017			0.0498 *	0.0498 *	0.1412	0.6217	0.0027 **
CTL 2017			0.6651	0.6651	0.0084 **	0.0051 **	0.0009 ***
OTC 2019			0.2031	0.2031	0.0480 *	0.0167 **	0.0200 **
CTL 2019			0.0052 **	0.0052 **	0.5372	0.3542	0.7229
Site1 2017	*t*-test *p*	OTC vs CTL	0.1438	0.1438	0.1157	0.0550	0.2014
Site2 2017			0.1019	0.1019	0.6441	0.3590	0.2955
Site1 2019			0.1106	0.1106	0.0452 *	0.0683	0.0757
Site2 2019			0.6952	0.6952	0.5535	0.7608	0.3536
Site 1 topsoil 2017	*t*-test *p*	OTC vs CTL	1.0000	1.0000	0.6397	0.3471	0.3114
Site 1 subsoil 2017			0.1201	0.1201	0.1450	0.1356	0.4442
Site 2 topsoil 2017			0.8075	0.8075	0.7021	0.6068	0.0468 *
Site 2 subsoil 2017			0.0533	0.0533	0.1826	0.0286 *	0.5745
Site 1 topsoil 2019			0.5286	0.5286	0.3631	0.3469	0.2681
Site 1 subsoil 2019			0.1835	0.1835	0.0889	0.1929	0.3078
Site 2 topsoil 2019			0.5286	0.5286	0.9181	0.8680	0.1504
Site 2 subsoil 2019			1.0000	1.0000	0.1040	0.8010	0.8588
Initial at Site 1	ANOVA *p*	2015, 2017, 2019	0.0944	0.0944	0.0116 *	0.0182 *	0.0147*
	Tukey *p*	2015 vs 2017	0.1849	0.1849	0.9992	0.6519	0.8250
		2015 vs 2019	0.9885	0.9885	0.0484 *	0.2022	0.0294 *
		2017 vs 2019	0.1247	0.1247	0.0152 *	0.0151 *	0.0329 *
Initial at Site 2	ANOVA *p*	2015, 2017, 2019	0.1537	0.1537	0.0916	0.0264 *	0.0886
	Tukey *p*	2015 vs 2017	0.5155	0.5155	0.1481	0.1092	0.1393
		2015 vs 2019	0.1382	0.1382	0.0880	0.0209 *	0.0866
		2017 vs 2019	0.5022	0.5022	0.9220	0.4998	0.9348

OTC, open-top chamber plots; CTL, control plots; *, $p < 0.05$; **, $p < 0.01$; ***, $p < 0 .001$.
